# Regulation of cancer epigenomes with a histone-binding synthetic transcription factor

**DOI:** 10.1038/s41525-016-0002-3

**Published:** 2017-01-09

**Authors:** David B. Nyer, Rene M. Daer, Daniel Vargas, Caroline Hom, Karmella A. Haynes

**Affiliations:** School of Biological and Health Systems Engineering, Arizona State University, 501 E Tyler Mall, Box 9709, Tempe, AZ 85287 USA

## Abstract

Chromatin proteins have expanded the mammalian synthetic biology toolbox by enabling control of active and silenced states at endogenous genes. Others have reported synthetic proteins that bind DNA and regulate genes by altering chromatin marks, such as histone modifications. Previously, we reported the first synthetic transcriptional activator, the “Polycomb-based transcription factor” (PcTF) that reads histone modifications through a protein–protein interaction between the polycomb chromodomain motif and trimethylated lysine 27 of histone H3 (H3K27me3). Here, we describe the genome-wide behavior of the polycomb-based transcription factor fusion protein. Transcriptome and chromatin profiling revealed several polycomb-based transcription factor-sensitive promoter regions marked by distal H3K27me3 and proximal fusion protein binding. These results illuminate a mechanism in which polycomb-based transcription factor interactions bridge epigenomic marks with the transcription initiation complex at target genes. In three cancer-derived human cell lines tested here, some target genes encode developmental regulators and tumor suppressors. Thus, the polycomb-based transcription factor represents a powerful new fusion protein-based method for cancer research and treatment where silencing marks are translated into direct gene activation.

## Cancer: Engineered protein switches on silenced genes

An engineered protein that manipulates DNA-protein packaging in cancer cells could offer a powerful new approach to treating tumors. Karmella Haynes and colleagues from Arizona State University in Tempe, USA, designed a protein that can recognize chemical tags on DNA-wound chromatin structures where gene activity is being silenced and then reactivate those epigenetically repressed genes. In previous work, Haynes’ team showed how this “Polycomb-based transcription factor” could stimulate gene expression at two silenced genes in one cancer cell line. Now, working in three different cancer cell types, the researchers demonstrate that treatment with this engineered protein increases the expression levels of many genes across the genome involved in developmental regulation and tumor suppression. If the engineered protein works specifically in cancer cells, while leaving healthy tissue alone, it could prove beneficial for patients.

## Introduction

Proteins from the gene regulatory complex known as chromatin mediate stable, epigenetic expression states that persist over multiple cell divisions in metazoan tissues. Harnessing the potent gene-regulating functions of chromatin proteins has become a high priority for cancer therapy and tissue engineering. The “histone code” model of chromatin function^1^ has strongly influenced work in epigenetic engineering and drug development.^2,3^ According to this model, biochemical marks are written onto DNA-bound histone proteins and these marks are read when effector proteins physically interact with the modified histones. Then the effector proteins enhance or inhibit transcription initiation. Much of our current understanding about chromatin-mediated gene regulation comes from deconstructive methods such as genetic mutations and RNA interference (for examples, see refs 4–6). Constructive approaches, where synthetic systems are built from chromatin components, are gaining recognition as an important and powerful research method^7^ as well as a powerful application for biomedical engineering.

**Fig. 1:**
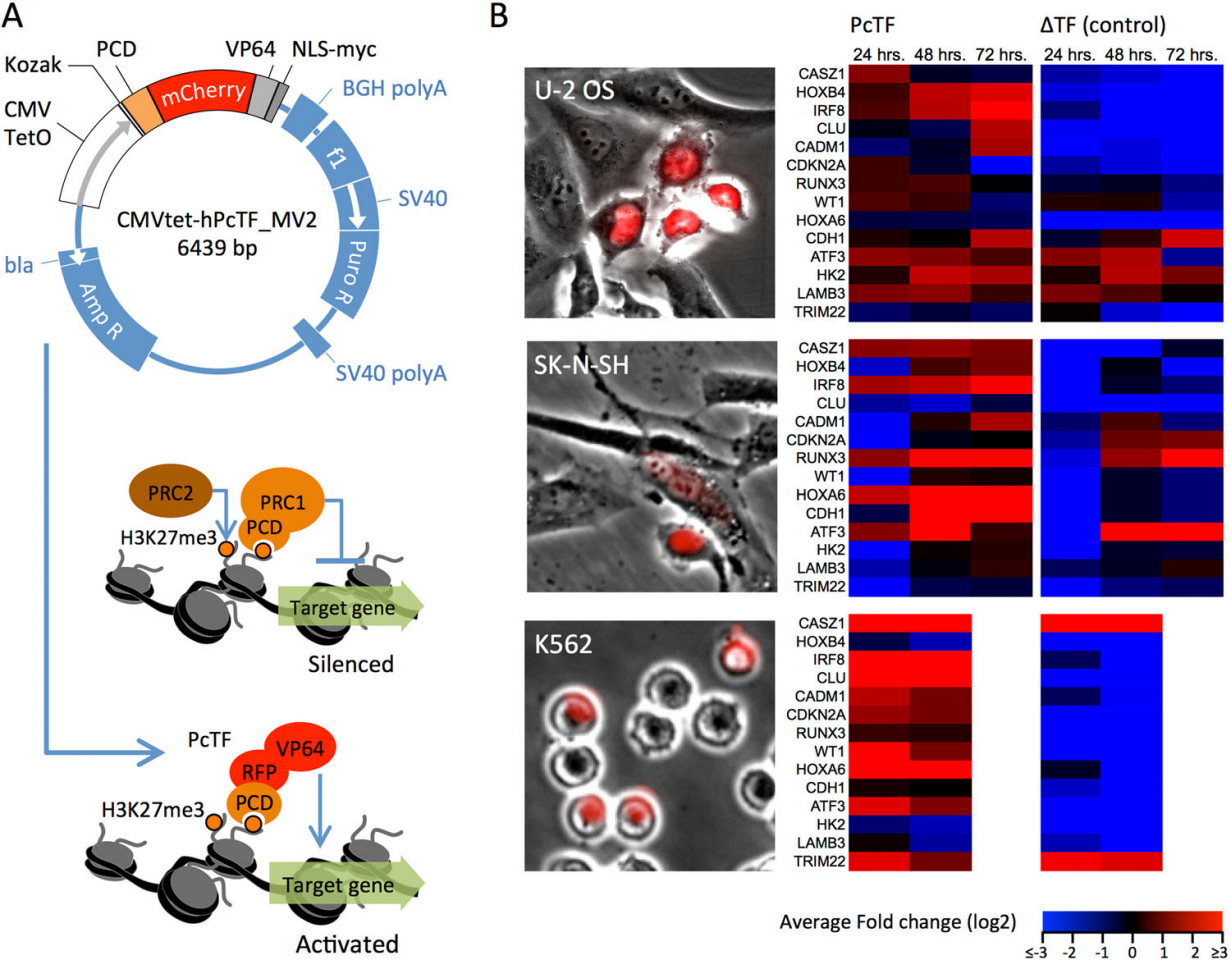
PcTF expression stimulates upregulation of known targets of Polycomb in transiently transfected cells. **a** Map of the PcTF-expressing plasmid (*top*). The natural PRC1 complex mediates gene silencing (*middle*). PcTF expression leads to accumulation of PcTF at H3K27me3 and gene activation (*bottom*).^11^ **b** Transiently-transfected U-2 OS, SK-N-SH, and K562 cells were visualized via the mCherry red fluorescent protein (RFP) tag. qRT-PCR was used to determine mRNA levels of fusion protein transcripts (PcTF or ΔTF) and a panel of 14 target genes at 24, 48, and 72 h post-transfection. RFP signal was not detected in K562 after 48 h, therefore later time points were omitted for K562 in this assay and other experiments. The heat map shows scaled, average log_2_ fold change ratios for *GAPDH*-normalized expression in plasmid-transfected cells compared to cells mock-transfected with the vehicle (Lipofectamine LTX) only. Standard deviations are shown in Fig. S1

We constructed a synthetic transcription factor based on the natural effector protein CBX8. In many human cancers, lysine 27 on histone H3 (H3K27) becomes marked by trimethylation at abnormally high levels near tumor suppressor loci. CBX8-containing complexes accumulate at H3K27me3 and repress gene transcription.^8^ A central step in the polycomb pathway is the specific interaction between the H3K27me3 mark and a hydrophobic binding pocket within the polycomb chromodomain (PCD) motif of CBX paralogs.^9,10^ We used this interaction to design a polycomb-based transcription factor (PcTF) that has VP64 (tetrameric VP16) and a visible red fluorescent tag (mCherry RFP) fused to the C terminus of the 60 amino acid PCD. The C-terminal VP64 domain allows PcTF to stimulate activation at repressed H3K27me3-associated genes (Fig. 1a). In previous work, we demonstrated H3K27me3-dependent PcTF activity at a model locus (*UASTk-luciferase*). PcTF also activated endogenous silenced loci in an osteosarcoma cell line (U-2 OS).^11^ Low-resolution protein mapping experiments at four loci identified two promoter regions that were co-occupied by PcTF and H3K27me3. Further investigation is needed to understand and accurately predict targets of the PcTF transcription activator. Here, we report genome-wide analyses that substantially advance our understanding of PcTF function. PcTF-stimulated gene activation in three different cancer cell types identified a large cohort of universally upregulated genes with a signature H3K27me3 enrichment profile. PcTF is enriched at transcription start sites within the nucleosome-free region of promoters and this profile depends upon the methyl-histone-binding PCD domain. We present a model where PcTF bridges distal H3K27me3 with endogenous transcription factors near the transcription start site. These findings provide significant progress towards predicting targets of PcTF based on distributions of epigenomic marks.

## Results

### Common polycomb-silenced loci are activated in PcTF-expressing cells

We measured PcTF-mediated activation of known polycomb targets in three different cell lines, osteosarcoma (U-2 OS), neuroblastoma (SK-N-SH), and leukemia (K562). We selected a panel of 14 genes for which polycomb-mediated silencing is supported by genetic and pharmacologic disruption studies^5,12–21^ and protein mapping studies^22–25^ in human cancers and stem cells: *ATF3*, *CADM1*, *CASZ1*, *CDH1*, *CDKN2A*, *CLU*, *HK2*, *HOXA6*, *HOXB4*, *IRF8*, *LAMB3*, *RUNX3*, *TRIM22*, and *WT1*. We transfected U-2 OS, SK-N-SH, and K562 cells with a PcTF-expressing plasmid (Fig. 1) via Lipofectamine LTX and allowed transfected cells to grow for 24, 48, and 72 h prior to further analysis. Flow cytometry showed that ~24–50% were RFP-positive 24 h post-transfection, and this number decreased roughly twofold every 24 h (Table S1). No RFP signal was detected in K562 at 72 h. Therefore, this sample was omitted from further experiments. We used quantitative reverse transcription PCR (qRT-PCR) to measure mRNA from mock-transfected (vehicle only) and plasmid-transfected cells (Fig. 1a). Primers and dye-conjugated hydrolysis probes are described in Table S2. We observed a decrease in PcTF transcripts over time (Fig. S1, Table S2) and therefore adjusted fold-change ratios based on initial PcTF transcript levels at 24 h (see Methods). In all three cell lines, over half of the genes were activated two-fold or higher compared to mock-transfected controls (Fig. 1b). Next, we carried out a control experiment to determine whether the histone-binding domain (PCD) was necessary for PcTF-mediated activation, as illustrated in Fig. 1a. To this end, we used delta-TF (ΔTF), a fusion protein that lacks the PCD histone-binding motif. Failure of ΔTF to upregulate genes in many cases demonstrated that the PCD was required to regulate a subset of target genes (Fig. 1b, Fig. S1). *IRF8*, *CADM1*, and *RUNX3* became activated in the presence of PcTF but not the PCD-deleted control in all three cell types. *CASZ1*, *HOXB4*, *CLU*, *CDKN2A*, *WT1*, and *HOXA6* were specifically upregulated in the presence of PcTF in two of the three cell types. Other genes that became activated in the presence of TF suggest a general stress-response to transfection, as observed in other work.^26^

In our previous work, we used an intercalating dye (SYBR green) for qRT-PCR and observed that *CDKN2A* was strongly upregulated by PcTF compared to ΔTF in U-2 OS cells.^11^ Here, our hydrolysis probe assay, which is more specific than the intercalating dye, shows modest upregulation (FC log_2_ = 0.68) of *CDKN2A* at the 24 h time point in U-2 OS and stronger upregulation in K562. *CDKN2A* is not upregulated in SK-N-SH. By including several more known polycomb targets, we have identified other genes that are specifically upregulated by PcTF in all three cell types: *IRF8*, *CADM1*, and *RUNX3*.

### PcTF stimulates expression of a large subset of H3K27me3-marked genes in U-2 OS, SK-N-SH, and K562 cells

**Fig. 2:**
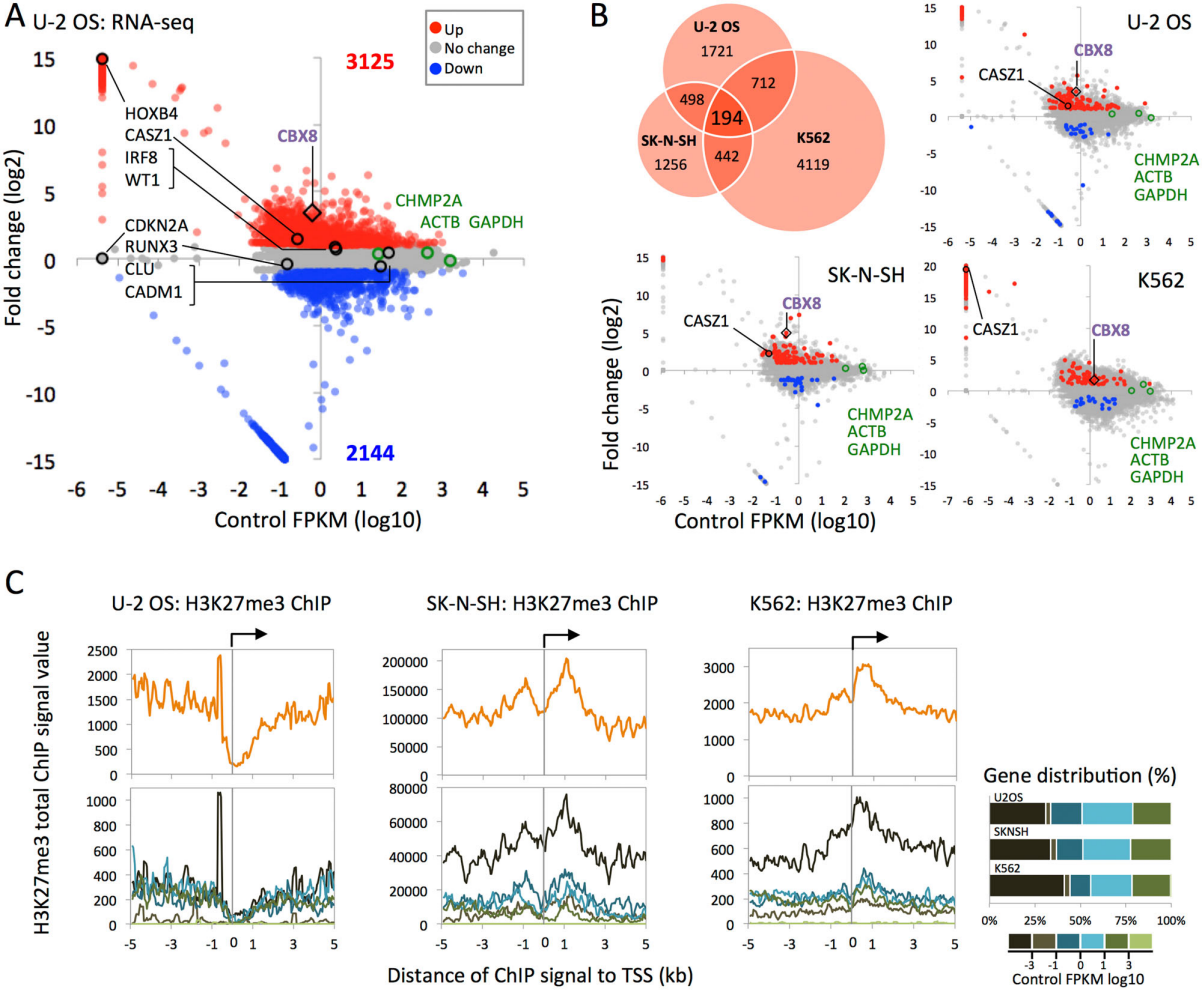
Genome-wide analysis of gene transcription and H3K27me3 at promoter regions in PcTF-expressing cells. **a** The scatter plot compares RNA-seq signals (FPKM log_10_) of 23,245 genes from cells that were mock transfected (Control) vs. log_2_ fold change of PcTF-expressing cells 96 h post-transfection for U-2 OS and SK-N-SH and 48 h for K562. Housekeeping genes *GAPDH*, *ACTB*, and *CHMP2A* are negative controls. **b** The Venn diagram shows unique and common sets of genes that became up-regulated at least twofold in the three cell types. Overall, 194 commonly up-regulated genes and 50 commonly downregulated genes are highlighted in the scatter plots. The PcTF-homologous *CBX8* gene was used as a proxy for PcTF expression levels. **c** TSS plots (*orange*, *top*) show total H3K27me3 ChIP enrichment values mapped at 200 bp intervals with a step value of 50 bp. In the *lower* plots, values are stratified by the basal gene expression level in untreated cells (Control FPKM log_10_). Stratified TSS-plot data were normalized by the gene distribution for each category

Whole-transcriptome analysis revealed a larger subset of PcTF-sensitive genes. We performed next-generation deep sequencing of total RNA (RNA-seq) from mock transfected (control) and PcTF-expressing cells (+PcTF) up to 96 h post-transfection. An increase in alignments to *CBX8*, which matches the first ~200 bp of the PcTF open reading frame, indicated expression of the PcTF transgene (Fig. 2a). Whole-transcriptome analysis of U-2 OS corroborated the qRT-PCR results for the PcTF-sensitive genes *CASZ1*, *HOXB4*, *IRF8*, and *WT1*. Comparison of fragments per kilobase of gene model per million mapped reads (FPKM) for control samples vs. fold change after PcTF expression showed that 13.4% of 23,245 annotated Refseq genes became up-regulated twofold or higher (Fig. 2a). *CASZ1* and 193 other genes showed upregulation in all three cell types (Fig. 2b).

We also observed down-regulation (fold-change ≤2) at 9.2% of the genes in U-2 OS. Fifty of these genes also became downregulated in SK-N-SH and K562. Repression is inconsistent with the transcriptional activation function of VP64. Based on the RNA-seq results alone, we speculated that the upregulated genes might be direct targets of PcTF (as illustrated in Fig. 1a) and that in some cases PcTF might up-regulate endogenous transcription regulators such as gene silencers that produce secondary effects. Seventy-six of the genes that are upregulated twofold or higher encode microRNAs, which silence gene expression by blocking translation of messenger RNA. MicroRNAs often have several targets, such as *MIR3658*, which has 126 target genes (miRTarBase)^27^ and is upregulated in U-2 OS and SK-N-SH. *MIR1248* has 154 target genes and is upregulated in SK-N-SH and K562. PcTF might also disrupt expression at the repressed genes by placing VP64 in a suboptimal position relative to the promoter.^28,29^

To investigate further, we used data from chromatin immunoprecipitation followed by deep sequencing (ChIP-seq) experiments to look for an epigenetic signature of PcTF-sensitive genes. We determined the enrichment of H3K27me3 at the 14 Polycomb targets (Fig. 1b) and near transcription start sites (TSS’s) of over 23,000 annotated human genes. In U-2 OS, ChIP was performed as previously described using an antibody against H3K27me3.^11^ DNA was purified from the immunoprecipitated chromatin, analyzed by next generation deep sequencing, and reads were aligned to the hg19 human genome consensus (Feb. 2009 GRCh37). Regions of enrichment compared to non-immunoprecipitated bulk chromatin (input) were calculated using the Hotspot algorithm.^30^ We used shared and public data to determine H3K27me3 enrichment in SK-N-SH and K562 cells. *CASZ1*, *IRF8*, and *CADM1* are marked by H3K27me3 (Fig. S2) and become upregulated upon PcTF expression in all three cell types. *CLU* and *HOXA6* are enriched for H3K27me3 in two cell types, and only become up-regulated when H3K27me3 is present (Fig. S2). These results are consistent with a mechanism where PcTF accumulates at H3K27me3 sites and induces gene transcription.

Overall, H3K27me3 enrichment was depleted near transcription start sites at silenced and active genes, indicated by a sharp valley at position zero in stratified TSS-plots (Fig. 2c). This profile has been observed in other reports; similar plots show a valley in the TSS profile at the nucleosome-free region (NFR) of promoters in mammalian cells.^31,32^ Genes with lower basal expression levels, prior to PcTF expression, show the highest levels of H3K27me3 enrichment, which is consistent with the role of H3K27me3 in gene silencing. Genes that became up-regulated in response to PcTF expression showed the highest H3K27me3 levels compared to non-responsive and down-regulated genes in K562 cells (Fig. S3). In SK-N-SH and U-2 OS however, up-regulated genes showed the same or lower H3K27me3 levels as non-responsive genes. Therefore, PcTF-sensitivity may not be determined by the mere presence of H3K27 methylation within 5 kb of the transcription start site. Instead, positioning of H3K27me3 relative to the TSS might distinguish PcTF-sensitive genes.

We hypothesized that a specific H3K27me3 distribution pattern around the TSS determines which genes are activated by PcTF. For a subset of 194 commonly upregulated genes H3K27me3 is depleted at the TSS, which is surrounded by signal peaks at intervals of roughly 1 kb (Fig. S3). The highest peaks for K562 were observed immediately downstream of the TSS and 3 kb upstream. These data suggest that PcTF-mediated activation of silenced genes may rely on H3K27me3 enrichment at positions distal to the TSS.

### ChIP analyses in stable transgenic U-2 OS cells shows PCD-dependent enrichment of PcTF near transcription start sites

**Fig. 3:**
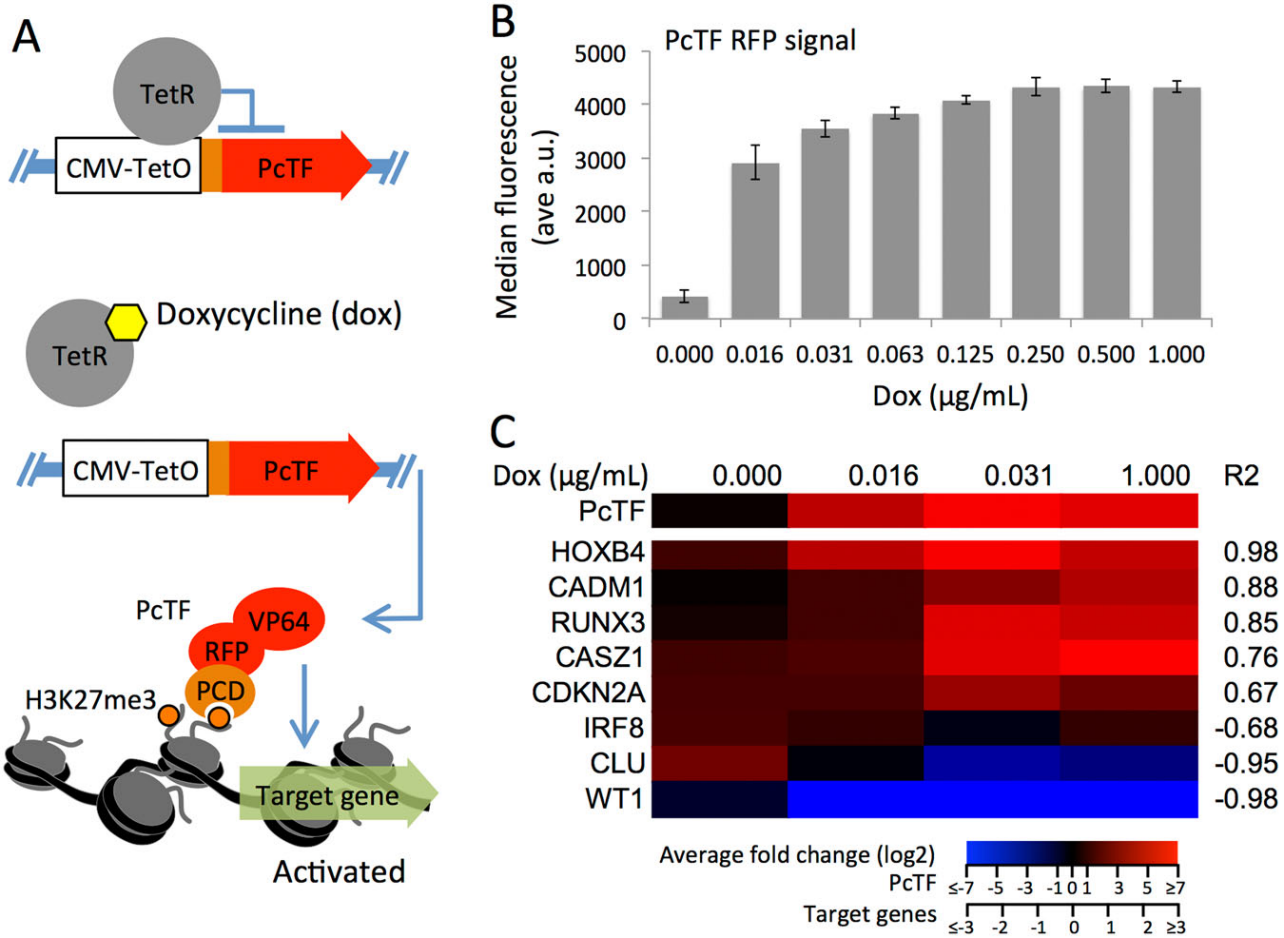
Polycomb-repressed genes become activated by PcTF in a dose-dependent manner. **a** The illustration shows doxycycline (dox) induced PcTF expression in U2OS-PcTF cells, leading to accumulation of PcTF at H3K27me3-positive promoters and gene activation.^11^ **b** Flow cytometry analysis of dox-treated cells. *Grey* bars show average RFP signal (*n* = 3, Error = standard deviation). RFP signal histograms and RFP-positive cell frequency are shown in Fig. S4. **c** qRT-PCR analysis of PcTF and eight genes that showed PcTF-specific activation in transiently transfected U-2 OS cells. The heat map shows average (*n* = 3) log_2_ fold change ratios for *GAPDH*-normalized expression in dox-treated cells compared to one of the untreated replicates. R2 = Pearson correlation coefficient of average log_2_ fold change values for target genes vs. PcTF

In order to further investigate the mechanism of PcTF-mediated gene regulation, we used crosslinked chromatin immunoprecipitation (X-ChIP, referred to as “ChIP” here) to measure the accumulation of PcTF and H3K27me3 at a single locus and throughout the genome. For these studies, we used an isogenic PcTF-expressing U-2 OS cell line. The cell line “U2OS-PcTF” carries a chromosomally-integrated, TetR-repressed gene that expresses myc-tagged PcTF when the cells are treated with doxycycline (dox) (Fig. 3a), as described previously.^11^ Five of the eight genes that we identified as specifically PcTF-sensitive (Fig. 1b) showed a dose-dependent response to PcTF in U2OS-PcTF cells. Flow cytometry of cells treated with none or 0.016–1 μg/ml dox for 72 h showed dose-dependent median RFP intensity (Fig. 3b). Frequencies of RFP-positive cells were the same across samples (Fig. S4), indicating that dox dosage regulated the amount of PcTF per cell. PcTF transcript levels also increased with dox dose, as shown by qRT-PCR (Fig. 3c). Expression of *HOXB4*, *CADM1*, *RUNX3*, *CASZ1*, and *CDKN2A* increased with higher levels of PcTF (Pearson *R* ^2^ = 0.67–0.98). *IRF8*, *CLU*, and *WT1* expression decreased with increasing PcTF, suggesting that perhaps regulation of these three genes is complex and may involve endogenous factors that oppose activation. Overall, our dose-response experiment validated U2OS-PcTF as a robust system to further investigate PcTF-mediated gene regulation.

**Fig. 4:**
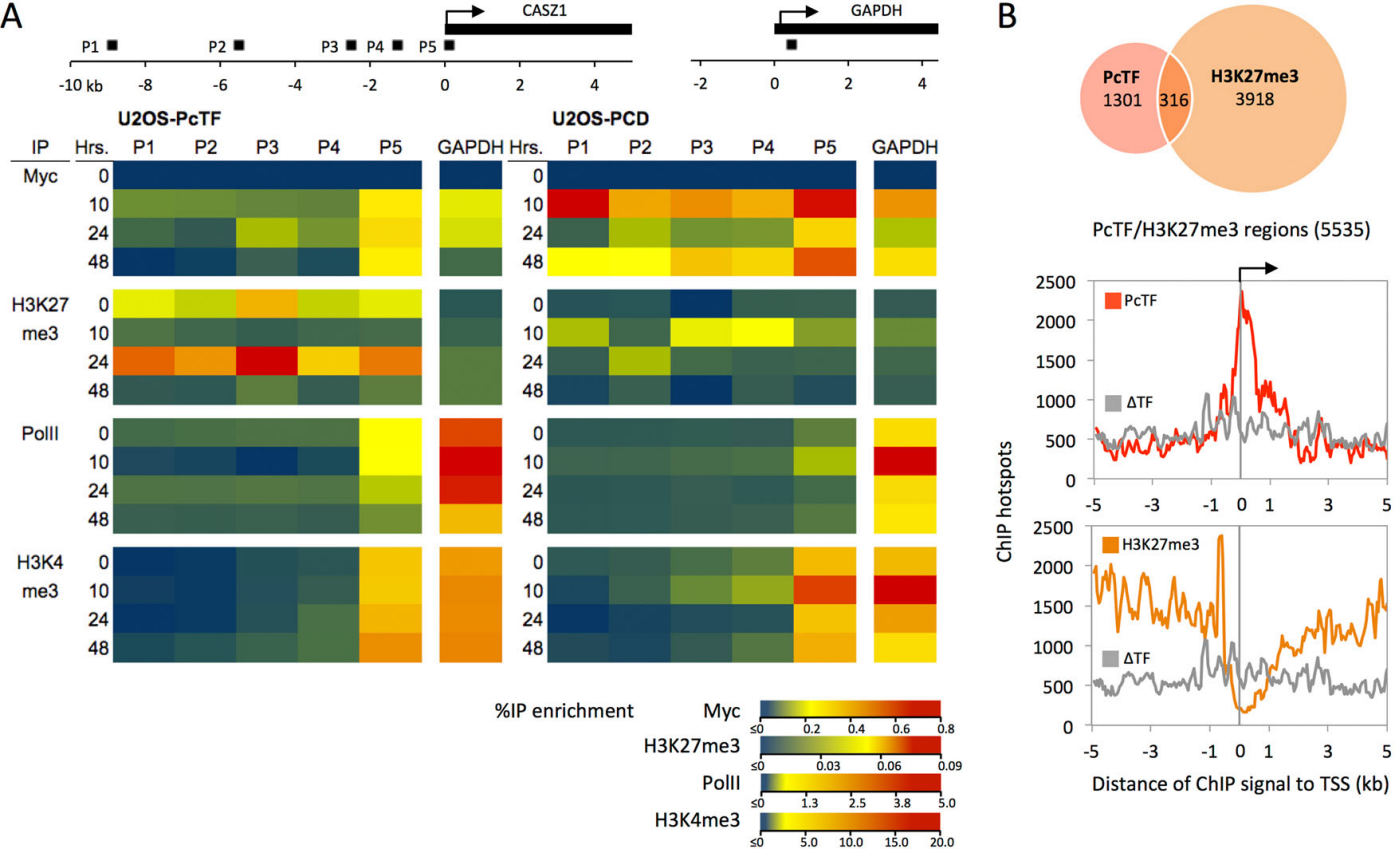
ChIP-PCR and ChIP-seq analysis of PcTF and H3K27me3 distribution in U2OS-PcTF cells. **a** The map shows the location of primer pairs and amplicons (Table S3) used to analyze DNA from IP-enriched chromatin. Heat maps show averages of triplicate qPCR reactions from duplicate (PolII, H3K4me3) or triplicate (Myc, H3K27me3) immunoprecipitations. Standard deviations are shown in the bar graphs in Fig. S6. **b** The Venn diagram compares genes that have PcTF- and H3K27me3-marked TSS regions (−5 to +5 kb). TSS-centered plots show the total ChIP hotspot signal value within a sliding window of 200 bp (step size = 50 bp)

In order to investigate the kinetics of PcTF engagement with a target gene, we measured accumulation of myc-tagged PcTF, H3K27me3, RNA PolII, and H3K4me3 (an activation-associated mark)^33^ at a PcTF-sensitive locus in U2OS-PcTF cells that were formaldehyde-fixed at different time points after dox treatment. A time course flow cytometry analysis of RFP in U2OS-PcTF cells confirmed PcTF accumulation over time from 2 to 10 h (Fig. S5). For ChIP analysis, we used *CASZ1* as a model locus. This gene was consistently upregulated by PcTF in the three cells types tested here, responded to PcTF in a dose-dependent manner in U2OS-PcTF, and the locus is free of other overlapping or nearby genes that might complicate analysis. We analyzed five sites by quantitative PCR (qPCR) of DNA from IP-enriched chromatin. These sites were located at the TSS and along 10 kb of the upstream region (Fig. 4a). A *GAPDH* TSS-proximal site was used as a negative control for PcTF and H3K27me3, and a positive control for H3K4me3 and PolII.

Prior to dox treatment (0 h), the *CASZ1* locus showed H3K27me3 enrichment ~ 2 kb upstream, and at lower levels at other sites, including the TSS (Fig. 4a, Fig. S6). PolII and the active H3K4me3 mark were also enriched at the TSS, suggesting that *CASZ1* is near a bivalent, poised promoter.^24^ After dox treatment, PcTF enrichment was observed near the TSS of *CASZ1* at 10, 24, and 48 h post induction. Over time, H3K27me3 levels fluctuated and then decreased at 48 h. In summary, we show that the H3K27me3 mark is present as PcTF accumulates at *CASZ1*. The results suggest that PcTF-mediated gene activation is followed by a loss of the H3K27me3 silencing mark.

We observed stronger PcTF enrichment at the TSS than at the strongest H3K27me3 signal 2 kb upstream, suggesting that PcTF binding might be controlled by interactions between VP64 and endogenous transcription factors rather than by the PCD domain. To investigate the function of the PCD we used a dox-inducible stable cell line, U2OS-PCD, which expresses a truncated version of PcTF that contained the PCD, mCherry, and the myc tag, but lacks the VP64 activation domain.^11^ The binding profile of PCD showed enrichment across the 10 kb region upstream of *CASZ1*, which overlapped with H3K27me3 10 h. after induction (Fig. 2b). This result suggests that the PCD motif is functional within the fusion protein. We therefore surmised that there is synergy between the function of the PCD domain, required for the activation of *CASZ1* and other genes, and the VP64 domain, required for enrichment near the TSS.

To further investigate the mechanism of PcTF-mediated gene regulation, we used ChIP-seq to compare the distribution of PcTF and H3K27me3 throughout the genome and at TSS regions. A parental U-2 OS cell line (Flp-in T-Rex, Invitrogen) was used to observe unaltered H3K27me3 patterns in the absence of PcTF. ChIP-seq analysis of an isogenic, dox-inducible U2OS-ΔTF cell line^11^ showed little overlap of ΔTF with PcTF and H3K27me3 (Fig. S7), which indicated that PcTF and H3K27me3 distributions were not due to random crosslinking. We analyzed the distributions of PcTF and H3K27me3 ChIP at different genomic features and observed the greatest enrichment over background for intergenic regions (Fig. S7). PcTF, but not H3K27me3, showed signal enrichment at promoters. These results indicate that PcTF and H3K27me3 co-occupy non-coding regions, and that PcTF is frequently enriched at promoter sites.

We inspected promoter regions more closely by identifying PcTF and H3K27me3-enriched regions surrounding TSS’s. We identified 5535 total regions that were marked with PcTF or H3K27me3 or both. A TSS plot showed a peak of PcTF enrichment at the TSS over a 2 kb interval (Fig. 4b). Randomly crosslinked ΔTF did not show the same enrichment profile, therefore mCherry-tagged VP64 alone does not account for the enrichment pattern of PcTF. In contrast to PcTF, H3K27me3 was depleted at the TSS. The subset of 316 regions in which we detected both PcTF and H3K27me3 showed a similar but shorter PcTF enrichment peak (Fig. S7). H3K27me3 signal was depleted near the TSS, with dispersed peaks of H3K27me3 further from the TSS. The profiling data suggest that at co-occupied regions, PcTF enrichment appears at and adjacent to the TSS, while H3K27me3 is farther away either upstream or downstream. Overall, these results suggest that PcTF accumulates near the TSS, and that this accumulation depends upon interaction with distal H3K27me3 through the N-terminal PCD peptide.

### Up-regulated genes are marked by H3K27me3 upstream and PcTF near the TSS in U2OS-PcTF cells

**Fig. 5:**
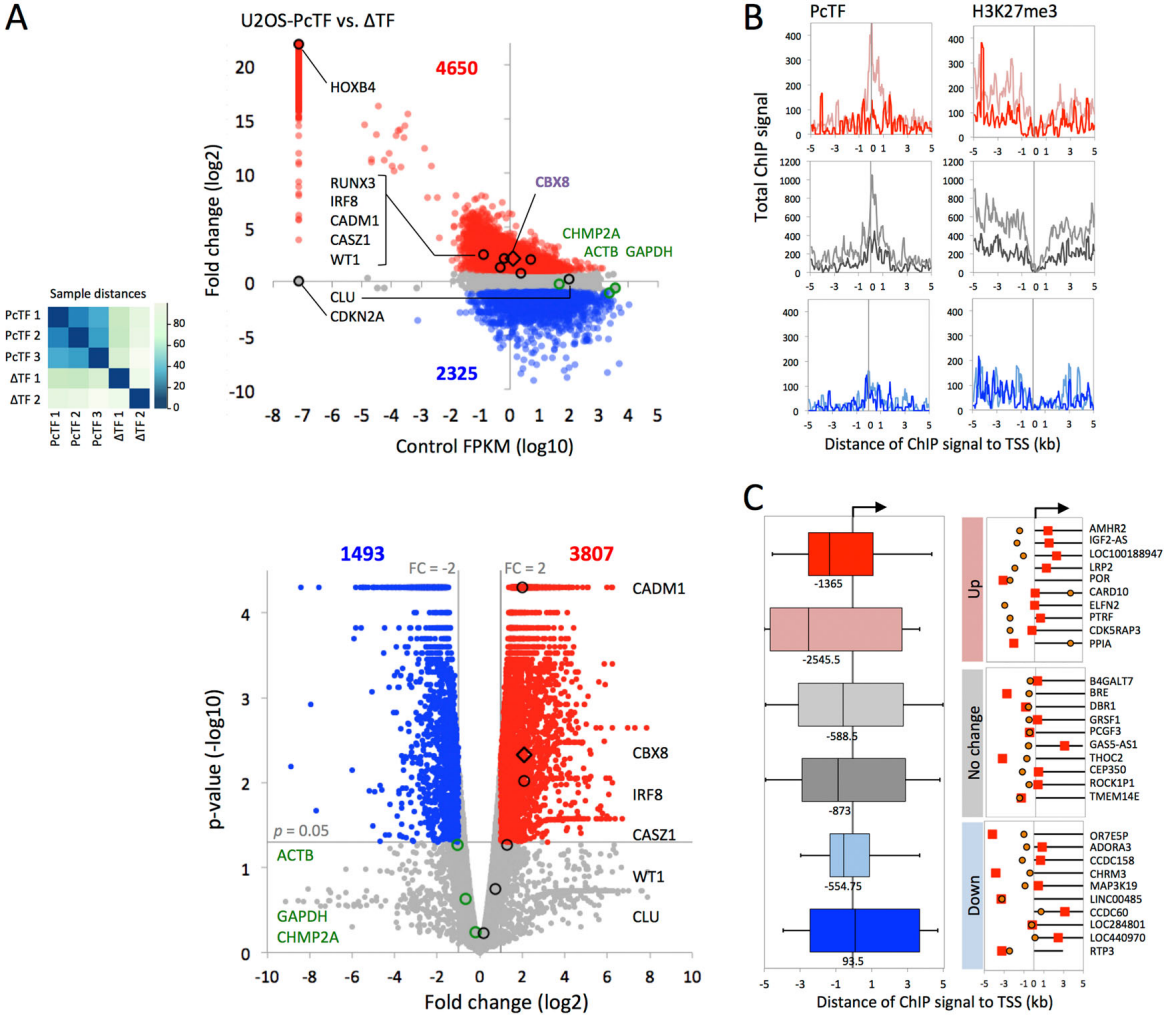
Analysis of genome-wide regulation in U2OS-PcTF cell and H3K27me3 localization. **a** The scatter plot compares RNA-seq signals (FPKM log_10_) of dox-induced ΔTF cells vs. the log_2_ fold change in expression compared to U2OS-PcTF cells (same color scheme as in Fig. 2a). Known Polycomb-regulated genes and control genes from Figs. 1 and 2 are highlighted for comparison with previous experiments. The volcano plot shows statistical significance vs. fold change for triplicate U2OS-PcTF samples. **b** TSS-centered plots show the total ChIP hotspot signal value (window = 200 bp, step size = 50 bp) stratified by log_2_ fold-change expression. **c** H3K27me3 ChIP signal distances from the TSS were compared for genes of lengths ≥2 kb in the subset of 316 genes where the 10 kb region is co-occupied by PcTF and H3K27me3. Genes were grouped by fold-change expression (as in **b**). Box plots show median values (*solid vertical line*), 25th (*left box*) and 75th (*right box*) percentiles, and minimum (*left whisker*) and maximum values (*right whisker*). TSS maps drawn to scale show the midpoints of ChIP signals for H3K27me3 (*orange circle*) and PcTF (*red square*) relative to the TSS. Genes are sorted from highest to lowest log_2_ fold-change value

Up-regulation of PcTF-sensitive genes corresponds with H3K27me3 enrichment at roughly 1–2 kb upstream of the transcription start site in regions where we also detected PcTF enrichment near the TSS. To identify up-regulated genes, we compared RNA-seq FPKM values of 23,245 genes for doxycycline-induced U2OS-PcTF cells and U2OS-ΔTF cells. Agreement between U2OS-PcTF RNA-seq results was confirmed by DEseq2 comparison of reads alignments per gene (Fig. 5a). Six of the specifically PcTF-activated genes identified previously (Fig. 1b) showed upregulation in the RNA-seq results: *IRF8*, *CASZ1*, *RUNX3*, *HOXB4*, and *WT1*. We observed a significant (*p* < 0.05) increase in expression for two of these upregulated genes, *IRF8* and *CADM1* (Fig. 5a). For transcript levels that changed at least twofold in either direction, the predominant response was an increase in expression of genes that had a low initial expression state in control cells. FPKM values of silenced genes are typically lower than 1.^34^ The frequency of upregulated genes within the non-expressing FPKM range of 10^−4^–10^0^ was 23%, whereas only 3% of active genes were upregulated (Fig. 5a).

Stratified TSS plots showed that PcTF was frequently enriched immediately downstream of the TSS at up-regulated genes (Fig. 5b). H3K27me3 was depleted near the TSS and showed peaks of enrichment 1 kb, 2 kb, and 4 kb upstream. Median distances of the nearest H3K27me3 signal were −2545.5 bp for 2- to 8-fold up-regulated genes and −1365 bp for genes that were up-regulated eightfold or higher (Fig. 5c). For genes that showed no change or were down-regulated, median distances ranged from −588.5 to 93.5 bp. Positioning of H3K27me3 very close to the TSS at non-responsive and down-regulated genes might interfere with VP64-driven gene activation, or cause VP64 to recruit transcription factors to a site that is not optimal for proper transcription. These data indicate that a distal H3K27me3 mark positions PcTF at an optimal position to allow the VP64 activation domain to stimulate gene transcription at target promoters.

### PcTF-sensitive loci are located near H3K27me3-marked promoters and repressed regions

Non-coding, intergenic regions play an important role in protein-mediated gene regulation. Many of these regions contain elements that show conservation of epigenetic marks across human cell types.^35^ In order to deepen our understanding of PcTF-sensitive sites, we investigated local chromatin features at genes that were upregulated, down-regulated or showed no change in response to PcTF in U-2 OS, SK-N-SH, and K562 cells. Investigation of the chromosomal positions of PcTF-sensitive genes, H3K27me3, and 15 classes of human chromatin states revealed that histone methylation near poised promoters and repressed regions corresponds with PcTF-mediated gene activation. Regions of interest were identified using the 15 classes of human chromatin states from the ENCODE project for nine different human cell types,^35^ including K562. Coordinates for elements of all 15 classes were extracted in tabular format from hg19 annotations from the public K562 cell data (UCSC Genome Browser). We identified the closest element upstream or downstream of 23,245 TSS’s and calculated median distances between the midpoint of each element and the TSS (Fig. 5a). Genes with FPKM values of zero in both control and treated cells were excluded from the calculations. Compared to non-responsive genes (log_2_ fold-change <2 in either direction), both up- and down-regulated genes were located near class 3 poised promoters, which are characterized by the presence of H3K27me3 and H3Kme4 and are thought to support gene state plasticity.^36^ Up- and down-regulated genes also showed greater proximity to class 12 repressed regions, which are typically enriched for Polycomb-associated marks such as H3K27me3. None of the chromatin classes we investigated showed a significant bias in TSS proximity specifically for the up-regulated genes. Our investigation of local chromatin features implicates poised promoters and Polycomb-repressed regions as factors for PcTF-sensitivity.

## Discussion

**Fig. 6:**
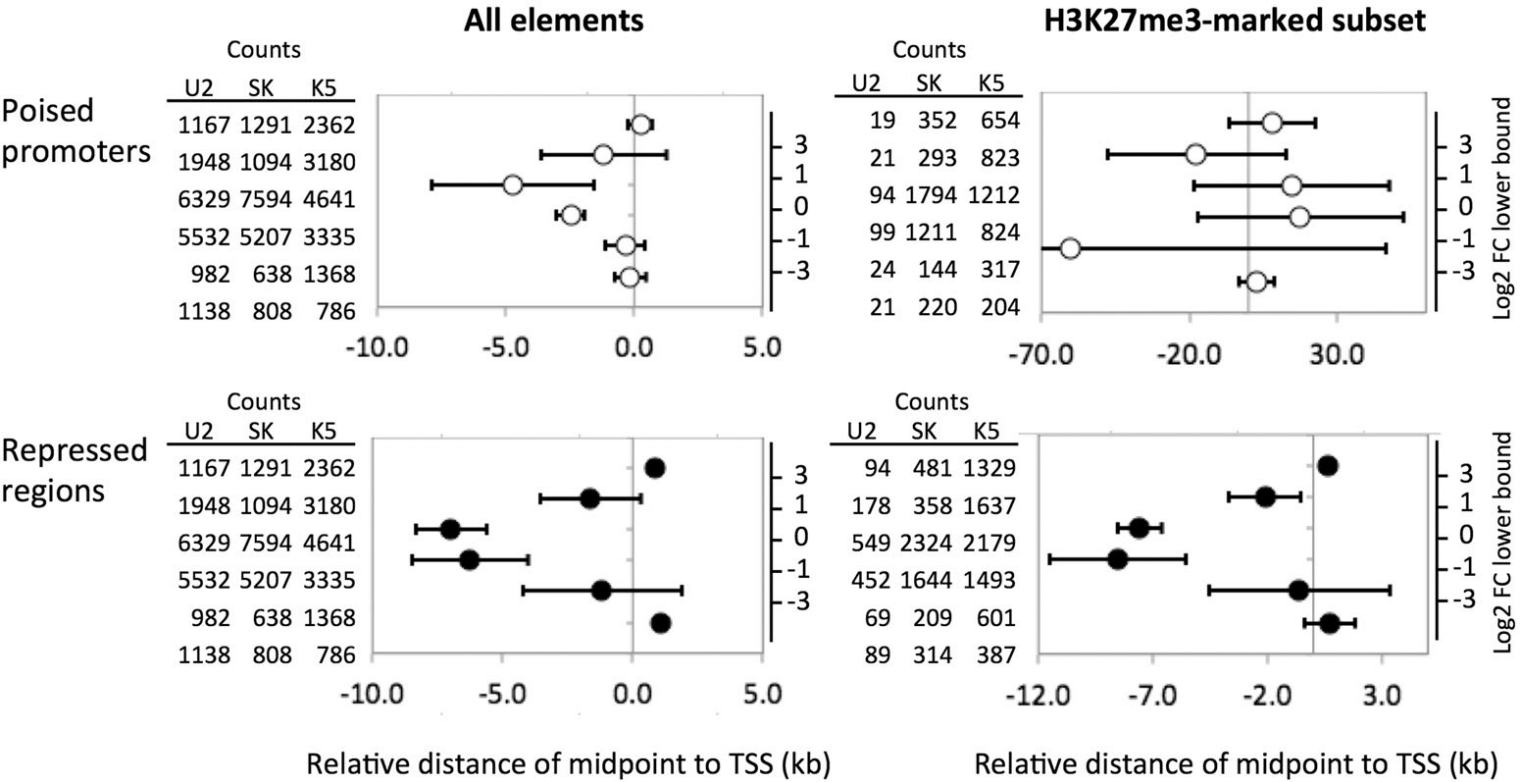
Distances of nearest chromatin states to TSS’s. Genes are stratified by fold-change in expression level after PcTF expression in transiently transfected cells. Numbers of regions nearest the TSS of genes in each category are shown in tables next to each chart. The coordinates of poised promoters, repressed regions, and 11 other states (not shown) were determined using chromatin state classes from the ENCODE project (UCSC Browser HMM track for K562 cells).^35^ U2 = U-2 OS, SK = SK-N-SH, K5 = K562

The work presented here provides new insights into the mechanism of PcTF, a synthetic chromatin-based transcription factor. PcTF couples recognition of a silencing-associated mark with gene activation. PcTF enrichment near TSS sites is similar to the patterns that have been observed for subunits of the transcription initiation complex,^31,37^ suggesting a strong interaction with endogenous transcription factors. The C-terminal domain of PcTF includes VP64, which is composed of four copies of a core acidic transcription activation domain (TAD) from VP16.^38^ The TAD is derived from the H1 region of VP16, which has been shown to bind with high affinity (high nanomolar range) to the mediator complex subunit MED25.^39^ The VP16 TAD also interacts with MED17, several members of the transcription factor II (TFII) family, and the TATA promoter motif-binding protein TBP (reviewed in refs 40,41). In contrast, the C-terminal PCD has relatively low affinity (high micromolar range) for H3K27me3.^42^ PCD-H3K27me3 binding may be weak *in vivo*, but it is still necessary for PcTF function^11^ and for TSS-proximal enrichment (Fig. 3c). Enrichment of PcTF in regions that are near to but do not overlap with H3K27me3 was observed in our previous ChIP-PCR studies.^11^ This offset of PcTF ChIP signals from H3K27me3 sites suggests that PcTF engages with H3K27me3 and then becomes trapped near the TSS through interactions with the transcription initiation proteins in crosslinked chromatin. The data provide strong evidence for a mechanism where PcTF bridges distal histone methylation marks with PolII-associated transcription factors at the nucleosome-free TSS (Fig. 6).

**Fig. 7:**
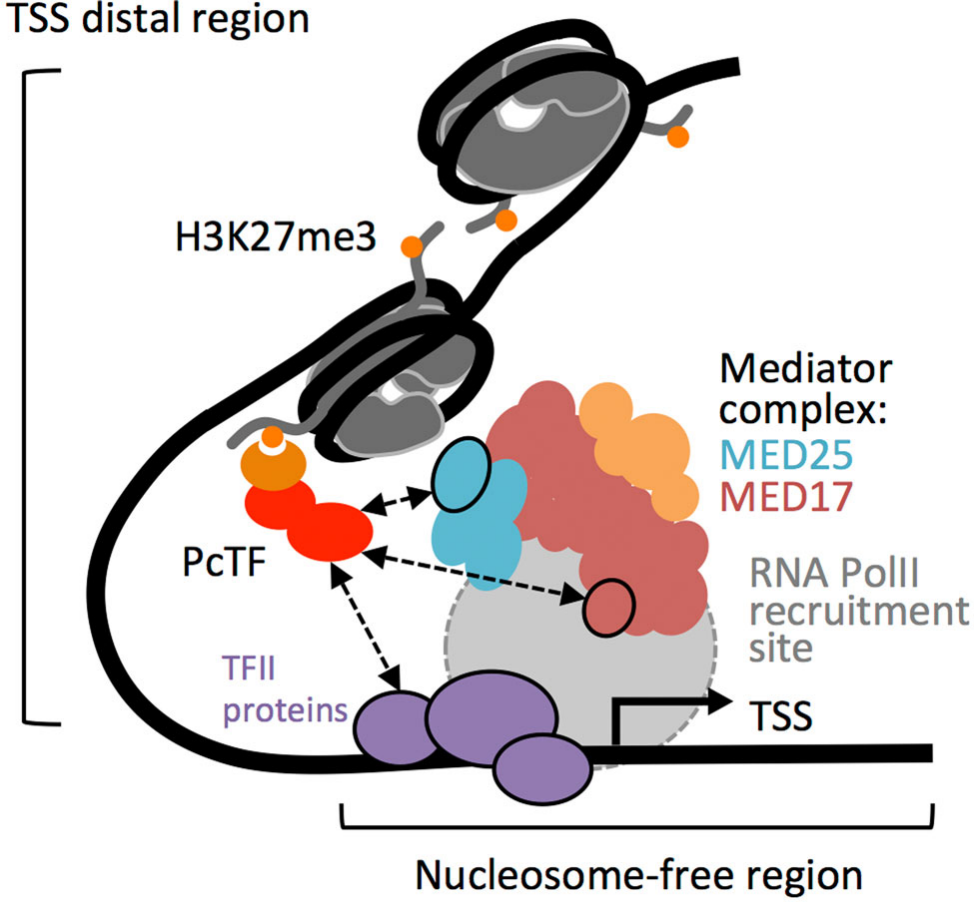
Model for PcTF-mediated gene activation.^57, 58^

RNA-seq results for genes that do not appear to be direct targets of PcTF-mediated activation provide additional insights into gene regulation (Fig. 7). Many of the genes we analyzed in the RNA-seq experiments do not change more than twofolds or become down-regulated after PcTF expression. In these cases, suboptimal positioning of the VP64 domain of PcTF relative to the promoter might neutralize its activity or lead to reduced expression below basal levels.^28,29^ We observed several genes that became up- or down-regulated, but lacked a PcTF signal within 10 kb of the TSS. Regulation of these genes could be mediated by transcription regulators that are direct targets of PcTF. For example up-regulated, PcTF-marked genes such as *CASZ1* may encode broadly-acting transcriptional regulators that control multiple targets.^43,44^

Our work has significant implications for medicine. Currently, epigenetic engineering relies largely on small-molecule inhibitors that are intended to erase cancer-associated epigenetic silencing and indirectly activate therapeutic genes. These drugs have major shortcomings such as incomplete erasure of cancer-associated methyl-histone marks and DNA damage. Here, we used a synthetic chromatin protein, PcTF, to convert the methyl-histone signal into direct activation of therapeutic genes instead of inhibitors to erase cancer-associated marks. Genes that were consistently upregulated by PcTF in all three cell lines we tested have therapeutic potential. Work by others has shown that overexpression of the *CASZ1* transcription regulator inhibits proliferation of neuroblastoma (SH-SY5Y).^45^ Ectopic expression of *IRF8* in renal cell carcinoma has been shown to decrease colony formation and migration.^46^ Overexpression of *CADM1* reduced proliferation in cervical cancer cell lines C33A, HeLa, SiHa, and CaSki.^47^ It will eventually be important to directly compare the efficacy of PcTF to RNAi knockdown or chemical inhibition (*e.g.* UNC3866) of Polycomb proteins.^12,48^ This comparison will determine whether PcTF-mediated stimulation of the transcription activation complex at silenced genes is truly advantageous over the simpler delivery of small molecules that inhibit polycomb and generate indirect activation. Furthermore, work to determine the specificity of PcTF in cancer vs. healthy cells and to enhance efficient synthetic protein delivery will bring this promising new fusion protein-based technology closer to translation.

## Methods

### DNA constructs

The doxycycline-inducible PcTF-expressing plasmid has been described.^11^ The plasmid is constitutively expressed in the absence of a TetR protein (in cell lines U-2 OS, SK-N-SH, and K562). The full annotated sequences of PcTF (hPCD-TF, KAH126) and ΔTF (TF, KAH132) are available online at Benchling—Hayneslab: Synthetic Chromatin Actuators (https://benchling.com/hayneslab/f/S0I0WLoRFK-synthetic-chromatin-actuators/).

### Cell culture and transfection

Complete growth media contained 10% tetracycline-free fetal bovine serum and 1% penicillin and streptomycin (pen/strep). U-2 OS (ATCC HTB-96), U2OS-PcTF, U2OS-PCD, and U2OS-ΔTF cells were cultured in McCoy’s 5A. K562 (CCL-243), and SK-N-SH (ATCC HTB-11) cells were cultured in IMDM or EMEM, respectively. Cells were grown at 37 °C in a humidified CO_2_ incubator. U2OS-stable cell lines were generated by previously published work.^11^ PcTF-expressing U-2 OS, K562, and SK-N-SH cells were generated by transfecting 5×10^5^ cells in 6-well plates with DNA/Lipofectamine complexes: 2 μg of plasmid DNA, 7.5 μl of Lipofectamine LTX (Invitrogen), 2.5 PLUS reagent, 570 µl OptiMEM. Transfected cells were grown in pen/strep-free growth medium for 18 h. The transfection medium was replaced with fresh, pen/strep-supplemented medium and cells were grown for up to 48 h (K562) or up to 96 h (U-2 OS and SK-N-SH).

### Quantitative reverse transcription PCR (qRT-PCR)

Total messenger RNA was extracted from ~ 90% confluent cells (~1–2×10^6^). Adherent cells (U-2 OS and SK-N-SH) were lysed directly in culture plates with 500 μl TRIzol. Suspended cells (K562) were collected into tubes, pelleted by centrifugation at 1000×*g* for 3 min at room temperature, separated from the supernatant, and lysed in 500 μl TRIzol. TRIzol cell lysates were extracted with 100 μl chloroform and centrifuged at 12,000×*g* for 15 min at 4 °C. RNA was column-purified from the aqueous phase (Qiagen RNeasy Mini kit 74104). SuperScript III (Invitrogen) was used to generate cDNA from 2 μg of RNA. Real-time quantitative PCR reactions (15 μl each) contained 1× LightCycler 480 Probes Master Mix (Roche), 2.25 pmol of primers (see Supplemental Table 1 for sequences), and 2 µl of a 1:10 cDNA dilution (1:1000 dilution for GAPDH and mCh). The real time PCR program was run as follows: Pre-incubation, ramp at 4.4 °C s^−1^–95 °C, hold 10 min; amplification, 45 cycles (ramp at 4.4 °C s^−1^–95 °C, hold 10 s, ramp at 2.2 °C s^−1^–60 °C, hold 30 s, single acquisition); cooling, ramp at 2.2 °C s^−1^–40 °C, hold 30 s. Crossing point (*C*_p_) values, the first peak of the second derivative of fluorescence over cycle number, were calculated by the Roche LightCycler 480 software. Expression level was calculated as delta *C*_p_, 2^[*C*_p__GAPDH_–*C*_p experimental gene_]. Fold change (log_2_) was determined as delta *C*_p transfected cells_/delta *C*_p mock_. Plasmid expression scaling for the values shown in Fig. 1b was performed using normalization quotients, calculated as average fold change (log_2_) for mCherry (plasmid expression) for the 24-, 48-, and 72 h time points divided by the 24 h value. Values for target genes within each time point were multiplied by the appropriate quotient.

### RNA-seq

RNA-seq was performed using one sample per experimental condition for transiently transfected cells and three replicates for the U2OS-PcTF and U2OS-ΔTF cell lines. Total RNA was prepared as described for qRT-PCR. Overall, 50 ng of total RNA was used to prepare cDNA via single primer isothermal amplification using the Ovation RNA-Seq System (Nugen 7102-A01) and automated on the Apollo 324 liquid handler (Wafergen). cDNA was sheared to ~300 bp fragments using the Covaris M220 ultrasonicator. Libraries were generated using Kapa Biosystem’s library preparation kit (KK8201). In separate reactions, fragments from each replicate sample were end-repaired, A-tailed, and ligated to index and adapter fragments (Bioo, 520999). The adapter-ligated molecules were cleaned using AMPure beads (Agencourt Bioscience/Beckman Coulter, A63883), and amplified with Kapa’s HIFI enzyme. The library was analyzed on an Agilent Bioanalyzer, and quantified by qPCR (KAPA Library Quantification Kit, KK4835) before multiplex pooling and sequencing on a Hiseq 2000 platform (Illumina) at the ASU CLAS Genomics Core facility.

### Bioinformatics analysis

ChIP-seq alignments were carried out using the Bowtie2 algorithm^49^ with the hg19 reference genome (Feb. 2009 GRCh37). Enrichments normalized to input were calculated using the Hotspot algorithm (distribution version 4).^31^ ChIP-seq data for SK-N-SH was provided by B. Bernstein and K562 data was retrieved from the UCSC Genome Browser website.^50^ Galaxy (http://www.usegalaxy.org)^51^ was used to identify overlaps with a minimum of 1 bp between gene intervals and ChIP-seq enrichment intervals. Raw sequence data (fastq) was analyzed with FastQC^52^ and processed to remove low-quality reads and adapters using TrimmomaticSE.^53^ RNA-seq alignments were carried out with de-multiplexed 50-bp single-end reads and the hg19 transcriptome. Within the public Galaxy online platform, splice junctions were mapped using Tophat (Galaxy version 2.1.0) and Bowtie 2 and transcript abundance and differential expression were calculated using Cuffdiff^54^ and the *Homo sapiens* UCSC hg19 gene transfer format (gtf) file from Illumina iGenomes. Out-dated genes were identified by cross-referencing gene symbols with the NCBI database and removed from the Cuffdiff output in Galaxy, resulting in 23,245 genes. Gene comparisons and Venn diagrams were generated with Venny 2.1 (http://bioinfogp.cnb.csic.es/tools/venny/)^55^ and Biovenn (http://www.cmbi.ru.nl/cdd/biovenn/).^56^ Microsoft Excel was used to calculate distances between features (TSS’s, ChIP signals, and enhancers) and to generate graphs and charts.

### Chromatin immunoprecipitation

Adherent U-2 OS cells were directly crosslinked in culture plates in 20 ml of 1% formaldehyde (Thermo Fisher Scientific)/1× PBS (Dulbecco’s) with gentle shaking for 10 min at room temperature. Cross-linking was stopped by adding 125 mM glycine, followed by 5 min gentle shaking. Quenched formaldehyde was aspirated and cells were washed twice for 5 min with gentle shaking at room temperature with 10 ml cold 1× PBS supplemented with Pierce Protease Inhibitors (Thermo Fisher Scientific). Cells were collected by scraping and spun at 200x*g* for 5 min. Cell pellets were washed twice with 10 ml cold 1× PBS with Pierce Protease Inhibitors. Overall, 70 μl of cross-linked cells were resuspended in 112.5 μl of cell lysis buffer (10 mM Tris pH 8 (ThermoFisher), 10 mM NaCl, 0.2% IGEPAL (Sigma)) plus Protease Inhibitors and incubated on ice 10 min. Lysed cells were spun for 5 min at 400x*g*. Nuclei were resuspended and lysed in 1 ml of nuclei lysis buffer (1% sodium dodecyl sulfate (SDS) (Sigma), 10 mM ethylenediaminetetracacetic acid (EDTA) (Fisher Scientific), 50 mM Tris–HCl pH 8.1 (Sigma)) plus Protease Inhibitors and incubated on ice for 10 min. Lysed nuclei were diluted with 0.5 ml of ChIP dilution buffer (1% Triton X-100 (Santa Cruz Biotech), 2 mM EDTA, 150 mM NaCl (Sigma), 20 mM Tris–HCl, pH 8) and split into five 300 μl-aliquots. Samples were sheared with the Qsonica Q700A Sonicator with a 5.5″ Cup Horn. Sonicated chromatin samples were spun at 16,300x*g* for 10 min at 4 °C to remove impurities and frozen at −80 °C. To determine shearing efficiency, DNA from 100 μl of each sample was purified by incubation at 65 °C in 100 mM NaCl overnight, at 37 °C with 10 μg RNase A (Sigma) for 30 min, and at 62 °C with 10 μg Proteinase K (Qiagen) for 2 h. DNA fragment sizes of ~500 bp were confirmed via electrophoresis on a 1% agarose gel.

Overall, 30 μg of each chromatin sample per immunoprecipitation was diluted to a final volume of 1 ml in ChIP dilution buffer. Magna ChIP Protein A + G Magnetic Beads (Millipore) were washed three times with PBS buffer + BSA (5 mg/ml) (Sigma). Chromatin samples were pre-cleared with 20 µl of washed beads and nutation for 3 h at 4 °C. Twenty percent of each pre-cleared sample was removed and set aside for input controls. Each chromatin sample was incubated at 4 °C for 12 h with nutation with each of the following antibodies: Anti-RNA PolII EMD Millipore 05-623, anti-H3K27me3 EMD Millipore 07-449, anti-H3K4me3 Abcam ab8580, anti-Myc (9B11) Mouse IgG Cell Signaling Tech 2276S, normal Mouse IgG Sigma 18765 (mock), and normal Rabbit IgG Cell Signaling Tech 2729S (mock). Magna ChIP Protein A + G beads were blocked by three washes in PBS buffer + BSA (5 mg/ml). Next, 20 μl of blocked beads were added to each antibody-chromatin and incubated for 3 h at 4 °C with nutation. Chromatin-antibody-bead complexes were washed twice for 10 min in RIPA buffer (50 mM HEPES pH 7.6 (Thermo Fisher Scientific), 1 mM EDTA, 0.7% sodium-deoxycholate (Sigma), 1% IGEPAL CA-630 (Sigma), 0.5 M LICl (Sigma)), twice for 10 min in a mild deteregent solution (20 mM Tris pH 8, 2 mM EDTA, 50 mM NaCl, 1% Triton X100, 0.1% SDS), and twice for 10 min in tris-EDTA pH 7.6 (Sigma). Elution of specifically bound chromatin was carried out in 100 μl of elution buffer (1% SDS, 0.1 M sodium bicarbonate (Sigma), 0.1 M NaCl). Inputs were thawed and brought to 100 µl with elution buffer. Samples were incubated for 30 min with nutation at room temperature. DNA was purified as described above, cleaned with a QIAquick PCR Purification Kit (Qiagen), and eluted in 50 µl nuclease-free water.

### Deep sequencing of DNA from immunoprecipitated chromatin (ChIP-seq)

Chromatin immunoprecipitation was performed on U-2 OS Flp-In T-Rex cells, which carry a chromosomal insert of a TetR-repressible PcTF gene as previously described.^11^ A ChIP-Seq DNA Sample Prep Kit (Illumina IP-102-1001) was used to prepare deep sequencing libraries from DNA that was purified from immunoprecipitated (IP) and non-IP (input) chromatin. End-repair was carried out at 20 °C for 30 min in the following 50 µl reaction: 30 µl ChIP DNA, 1× T4 DNA ligase buffer, 0.4 mM dNTP, 1 µl T4 DNA polymerase, 1 µl Klenow DNA polymerase, 1 µl T4 polynucleotide kinase. End-repair products were concentrated into a final volume of 34 µl Qiagen elution buffer (QIAquick PCR Purification kit 28104). 3′-end adenine base extension was carried out at 37 °C for 30 min in the following 50 µl reaction: 34 µl end-repair reaction product, 1× Klenow buffer, 0.2 mM dATP, 1 µl Klenow exonuclease. Base extension products were concentrated into a final volume of 10 µl H_2_O (Zymo Clean and Concentrator D4003). Adapter ligation was carried out at room temperature for 15 min in the following 30 µl reaction: 10 µl 3′-base extension reaction product, 1× ligase buffer, 1 µl adapter oligo mix, 4 µl DNA ligase. Ligation products were concentrated into a final volume of 10 µl H_2_O (Zymo Clean and Concentrator D4003). A total of 150–200 bp fragments from each ligation reaction were resolved and purified via gel electrophoresis and extraction (Zymoclean Gel DNA recovery kit D4001). DNA was back-eluted from the column twice with 10 µl H_2_O and brought to a final volume of 36 µl. Adapter-modified 150–200 bp fragments were enriched by the PCR in the following 50 µl reaction: 36 µl gel-purified DNA, 1× Phusion buffer, 0.3 mM dNTP, 1 µl PCR primer 1.1, 1 µl PCR primer 2.1, 0.5 µl Phusion polymerase. The cycling program was 98 °C/30 s, 18 cycles (98 °C/10 s, 65 °C/30 s, 72 °C/30 s), 72 °C/5 min, 4 °C/∞. PCR products were concentrated into a final volume of 15 µl H_2_O (Zymo Clean and Concentrator D4003). Libraries were size-confirmed on an Agilent 2100 Bioanalyzer and subjected to deep sequencing with single-end 100 bp reads on an Illumina Hi-Seq SR flow cell platform.

### Quantitative PCR of DNA from immunoprecipitated chromatin (ChIP-PCR)

ChIP samples were analyzed using real-time quantitative PCR in 15 μl reactions containing 7.5 μl SYBR Green master mix, 2.25 pmol of primers, and 2 μl of IP, IgG-IP (mock), or input template DNA. The real time PCR program was run as follows: pre-incubation, ramp at 4.4 °C s^−1^–95 °C, hold 10 min; amplification, 45 cycles (ramp at 4.4 °C s^−1^–95 °C, hold 10 s, ramp at 2.2 °C s^−1^–60 °C, hold 30 s, single acquisition); cooling, ramp at 2.2 °C s^−1^–40 °C, hold 30 s. To account for sampling 20% of the chromatin prep, input crossing point (*C*_p_) values were adjusted by subtracting log_2_(20). % IP DNA was calculated as 100 × 2^[C_p input_ - C_p IP_]. %Mock-IP DNA was calculated as 100 × 2^[C_p input_ - C_p mock_] and subtracted from % IP DNA to determine % IP DNA enrichment relative to mock-IP.

## Electronic supplementary material

Supplemental Figures and Tables
