# Erratum: Regulation of cancer epigenomes with a histonebinding synthetic transcription factor

**DOI:** 10.1038/s41525-017-0029-0

**Published:** 2017-09-05

**Authors:** David B. Nyer, Rene M. Daer, Daniel Vargas, Caroline Hom, Karmella A. Haynes

**Affiliations:** School of Biological and Health Systems Engineering, Arizona State University, 501 E Tyler Mall, Box 9709, Tempe, AZ 85287 USA

**Erratum to:** *npj Genomic Medicine* (2017); doi:10.1038/s41525-016-0002-3; Published 09 January 2017

The following text was erroneously included in the final publication (first paragraph of the Introduction, page 1): “Since the references were not in order it is has been rearranged it”. This text has now been deleted in the HTML and PDF versions of this article.

