# Supplementary material for: Regulation of cancer epigenomes with a histone-binding synthetic transcription factor
Source: NPJ Genom Med. 2017 Jan 9;2:1. doi: 10.1038/s41525-016-0002-3 (PMC5600530; doi:10.1038/s41525-016-0002-3)
Supplement: Supplementary file 1 — Supplemental Figures and Tables [file 41525_2016_2_MOESM1_ESM.docx]

SUPPLEMENTAL FIGURES


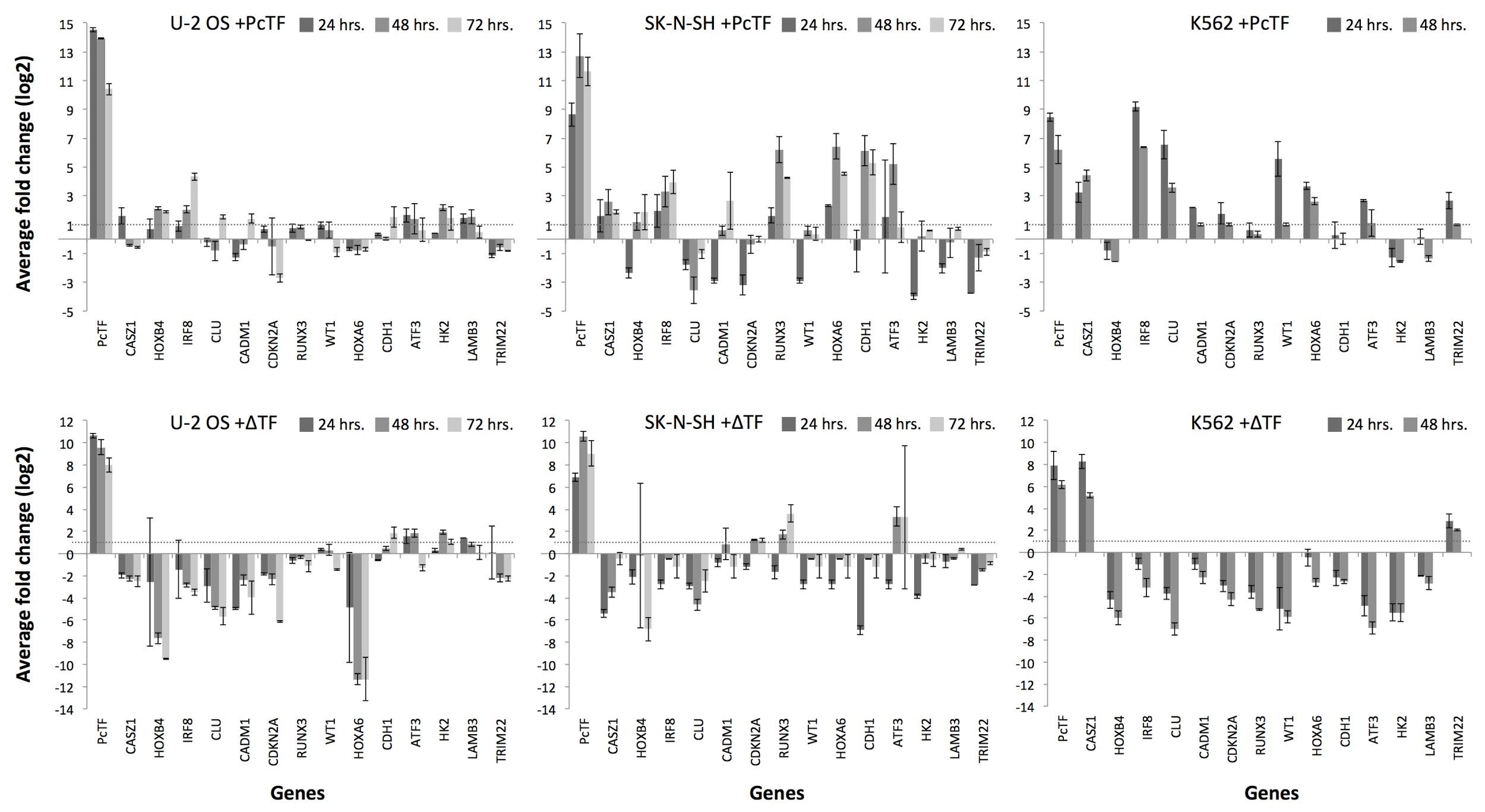


**Figure S1**. Bar chart representation of the qRT-PCR data from Figure 1. qRT-PCR was used to determine mRNA levels of PcTF (A) or ΔTF (B) and a panel of 14 target genes 24, 48, and 72 hours post-transfection. RFP signal was not detected in K562 after 48 hours, therefore later time points were omitted for K562 in this assay and in other experiments. Bars represent average log2 fold change ratios for GAPDH-normalized expression in plasmid-transfected cells compared to cells mock-transfected with the vehicle (Lipofectamine LTX) only. Error bars = standard deviation for two independent transfections.


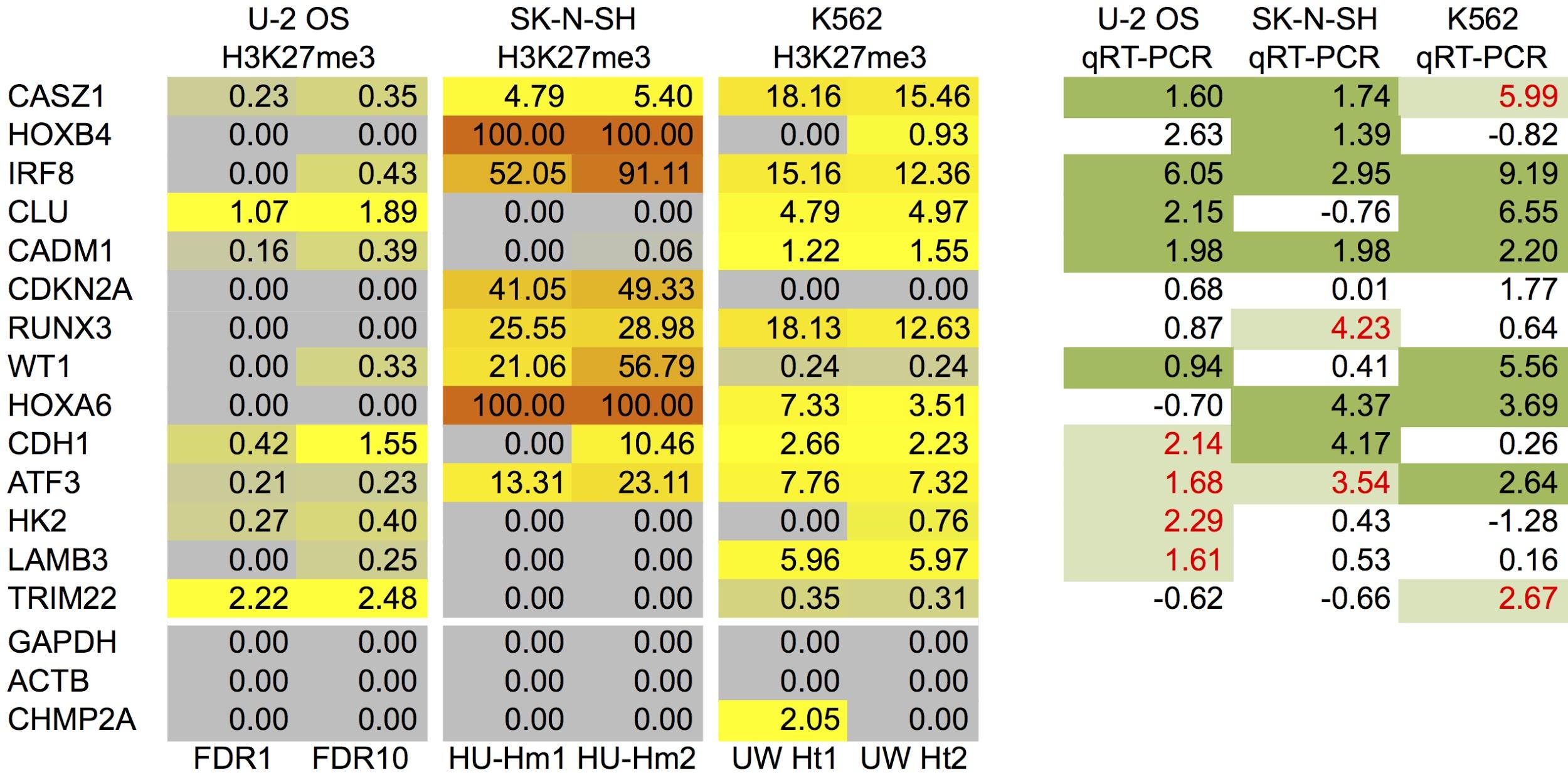


**Figure S2**. H3K27me3 enrichment at known Polycomb-regulated genes in U-2 OS, SK-N-SH, and K562 compared to gene response in the presence of PcTF (qRT-PCR data from Figure 1). H3K27me3 ChIP enrichment data for 14 known Polycomb-repressed genes and one active housekeeping-gene control (GAPDH). For each gene, we calculated H3K27me3 ChIP signal coverage over the gene body and 1000 bp upstream of the TSS. For SK-N-SH and K562, we assessed the level of histone H3K27 trimethylation using shared and public data. SK-N-SH signals were determined using the Homer algorithm^1^ for two replicate experiments (HUHm1 and HUHm2). SK-N-SH data was provided by B. Bernstein prior to submission to UCSC ENCODE database. K562 signals were determined using the Hotspots algorithm (UCSC Accession wgEncodeEH000923) for two replicate experiments (UWHt1 and UWHt2). For U-2 OS Hotspot values, FDR1 = a false discovery rate of 1%, FDR10 = a false discovery rate of 10%. The qRT-PCR columns (left) show the maximum (over the time points tested) scaled log2 fold-change value in PcTF-expressing cells for each gene shown in Figure 1b. Green highlighted cells indicate H3K27me3-positive genes that are also activated (rounded log2 fold-change ≥ 1.0) in the presence of PcTF. Cases where genes were non-specifically activated by the ΔTF control protein are highlighted in red font.


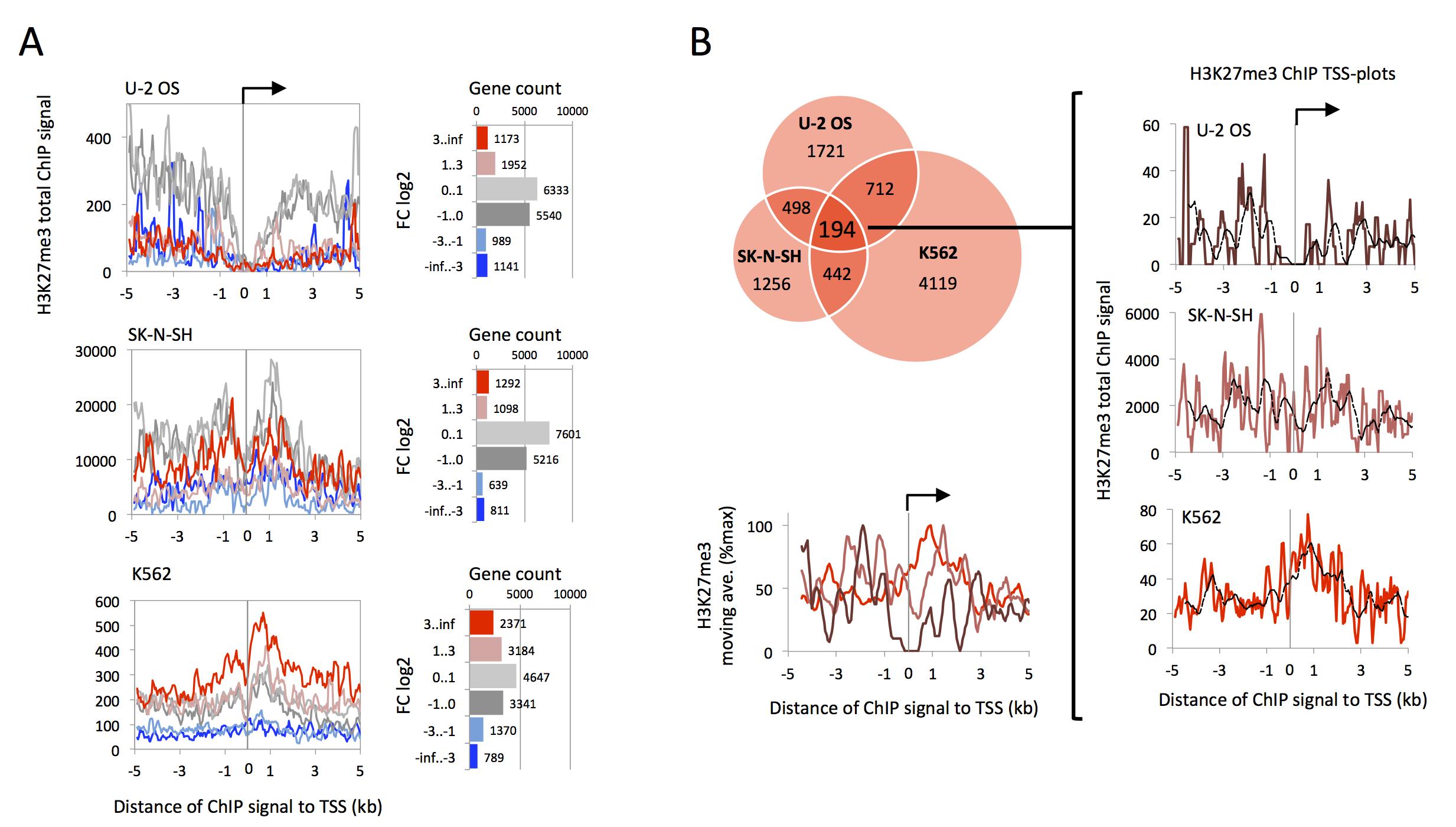


**Figure S3**. TSS plots show total H3K27me3 ChIP enrichment values mapped at 200 bp intervals with a step value of 50 bp. (A) Values are stratified by gene expression level (FPKM log10) by fold change (FC log2) after PcTF treatment and normalized by the frequency of genes in each category. Genes where FPKM = 0 for both control and treated cells were excluded from the FC log2-stratified data. (B) Moving averages for each TSS plot (dashed line, window = 10) are scaled and overlaid in the bottom plot.


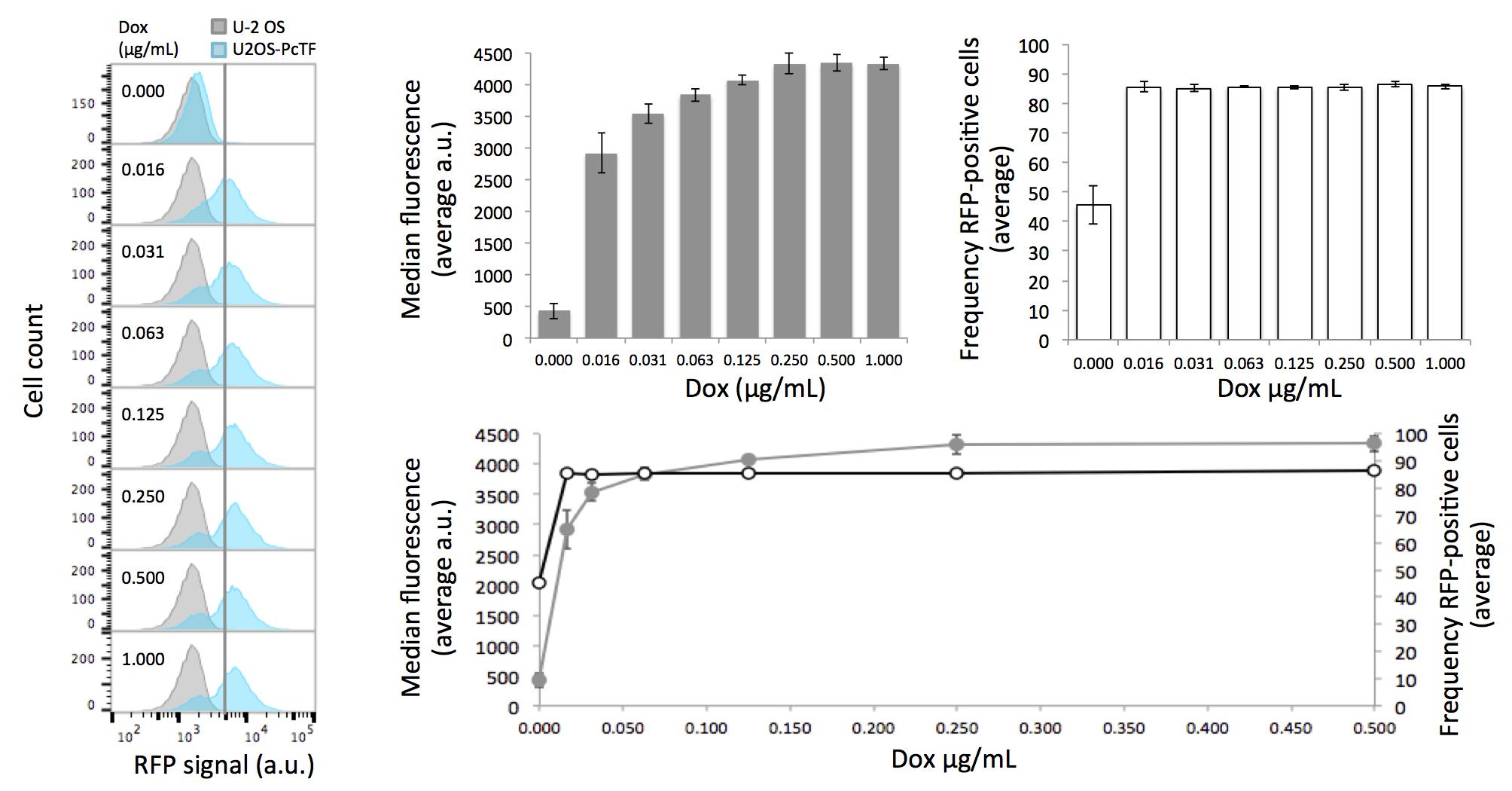


**Figure S4**. Flow cytometry analysis of U2OS-PcTF cells treated with varying concentrations of doxycycline. Cells were harvested by standard trypsinization (see Methods), resuspended in 1xPBS supplemented with 1% FBS, and analyzed on a BD Accuri C6 flow cytometer using CFlow Plus software. The filter used to measure RFP was 675 nm LP. Histograms (left) show cell counts vs. RFP signal (a.u.) for “blank” U-2 OS cells (no transgene) and U2OS-PcTF cells. U-2 OS cells were used to gate RFP-positive populations. The grey line at 1.0x10^5^ a.u. is a reference mark to visualize the shift in the RFP peak as dox concentration increases. Median RFP signals and RFP-positive cell frequencies were measured for triplicate samples. Error = standard deviation. The dot and line plot compares the change in median RFP signal (grey) to the change in RFP-positive cell frequency (white) over dox concentration.


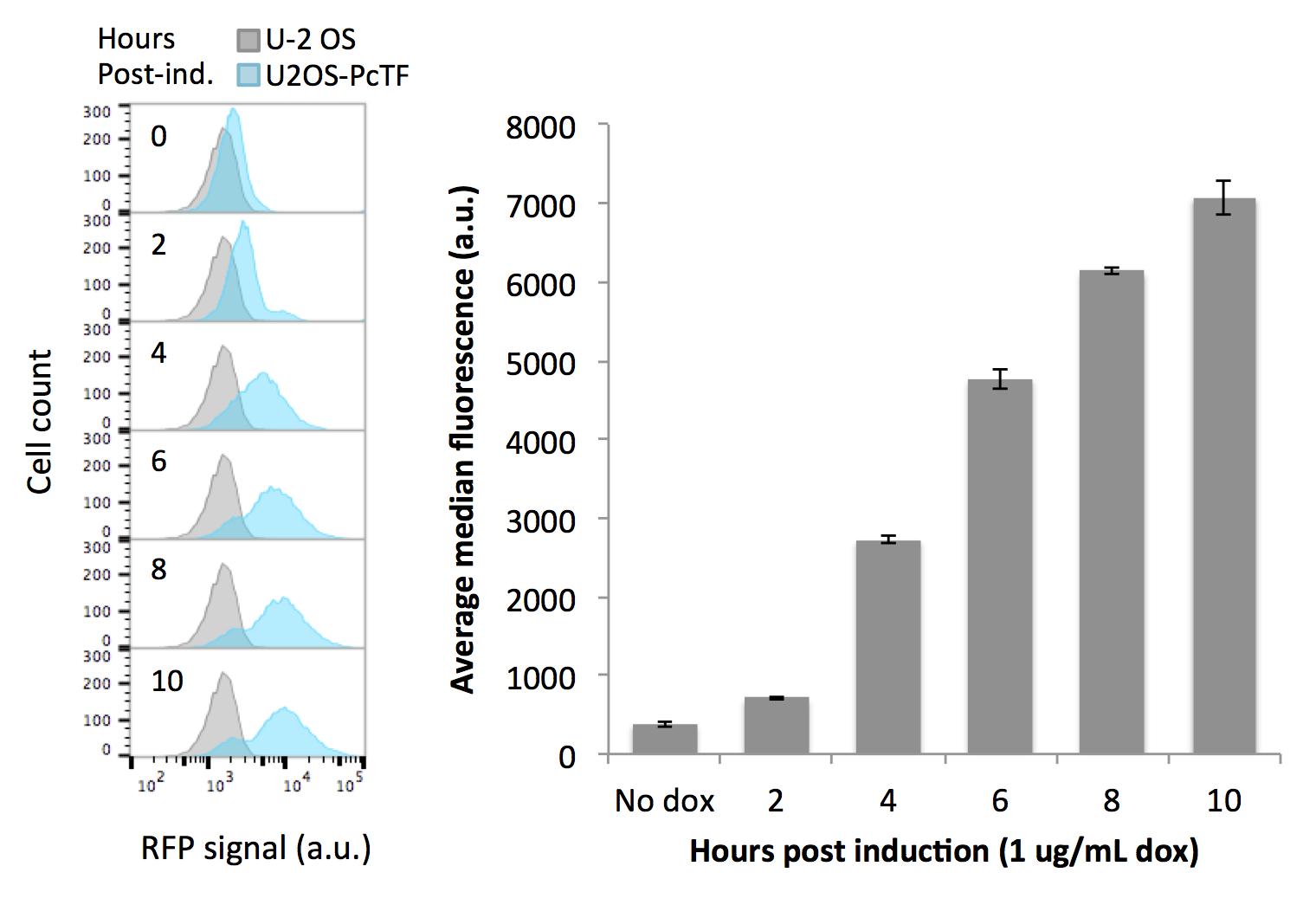


**Figure S5**. Flow cytometry analysis of the change in RFP signal over time in U2OS-PcTF cells treated with 1 μg/mL doxycycline. The assay was carried out as described in Figure S4, accept that cell samples were either untreated (0 hrs.) or induced at 2 hour intervals, grown for two additional hours, and then harvested for flow cytometry. Median RFP signals were measured for triplicate samples. Error = standard deviation.


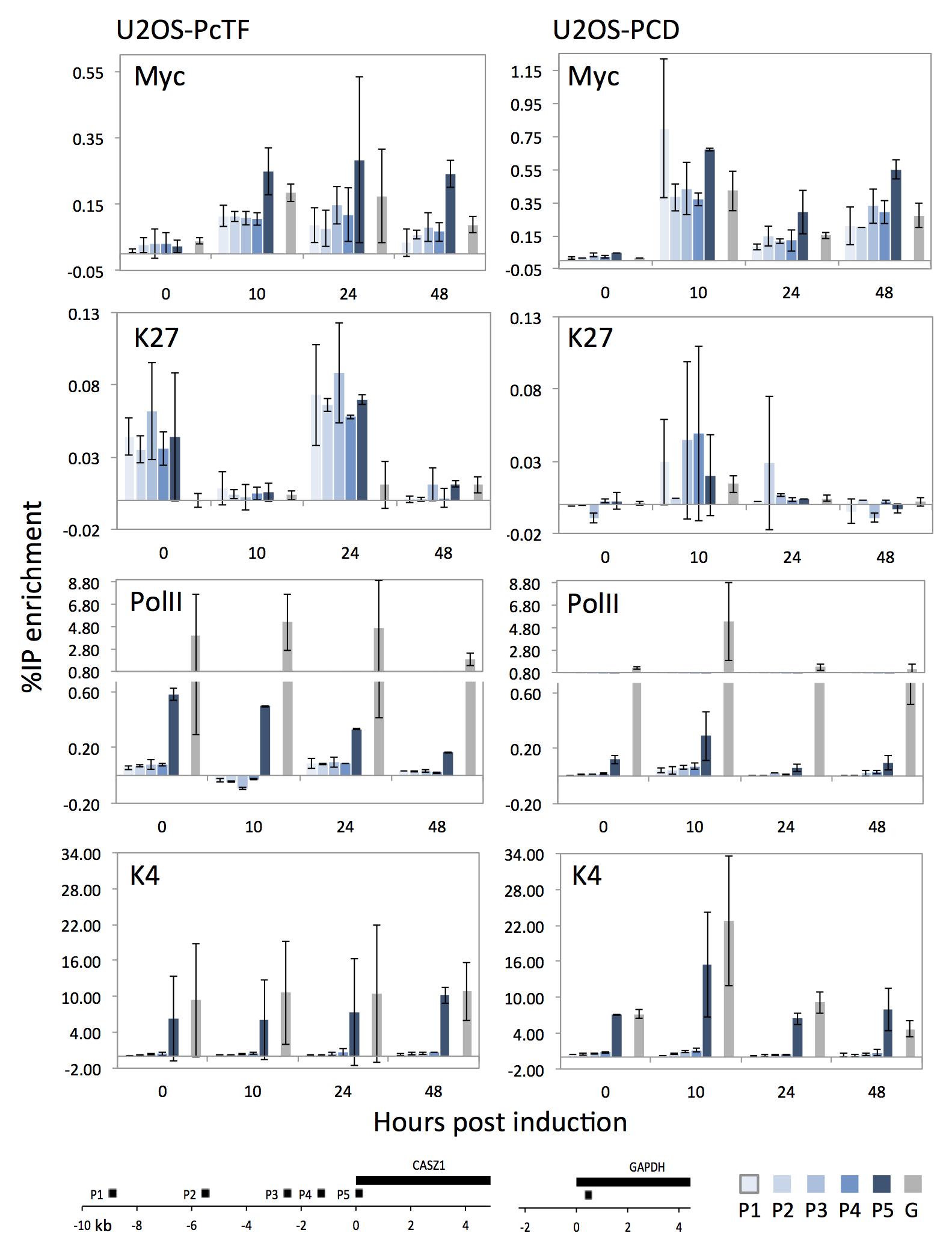


**Figure S6**. Bar chart representation of the ChIP-PCR data from Figure 4. Bars are means of triplicate qPCR reactions from duplicate immunoprecipitations for high-signal data (PolII, K4) and triplicate IP’s for low-signal data (Myc, K27).


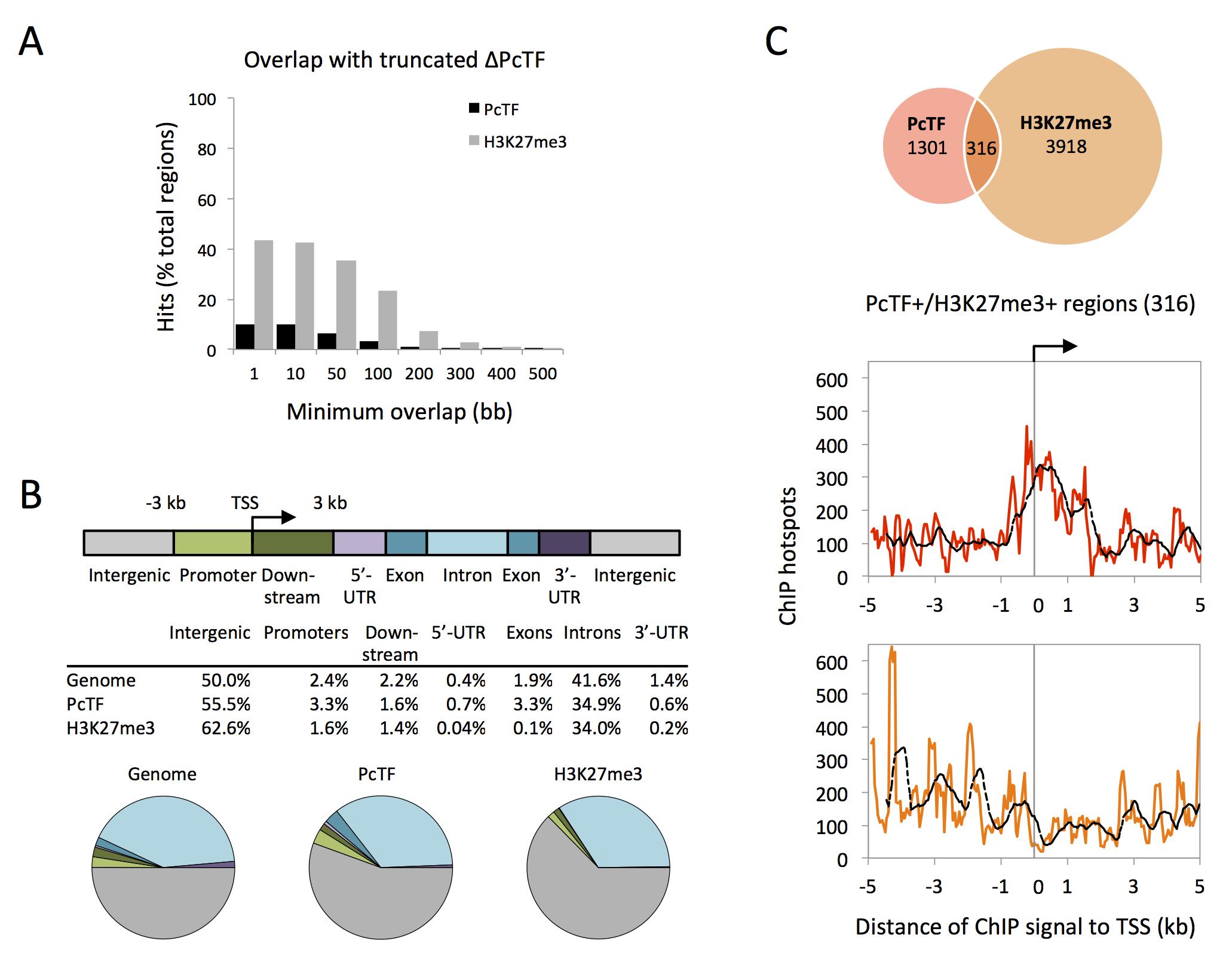


Figure S7. (A) Overlap of PcTF and H3K27me3 ChIP-seq signal regions (Hotspots, FDR 1%) with signal regions from ChIP-seq for a cell line that expresses a truncated version of PcTF that lacks the PCD (histone-binding domain). (B) Distribution of PcTF and H3K27me3 ChIP signals (hotspots) in genes and intergenic regions compared to background (Genome) distributions. ChIP-seq profiling across genes and intergenic regions was performed using the Cistrome platform^2^. (C) The venn diagram compares genes that have PcTF- and H3K27me3-marked TSS regions (-5 kb to +5 kb). The TSS-plots show the subset of 316 genes where the TSS region is co-occupied by PcTF and H3K27me3. Dashed lines show the moving average (window = 10).

SUPPLEMENTAL TABLES

|  | PcTF plasmid | | | ΔTF plasmid | | |
| --- | --- | --- | --- | --- | --- | --- |
| Cell line | 24 hrs. | 48 hrs. | 72 hrs. | 24hrs. | 48 hrs. | 72 hrs. |
| U-2 OS | 50.4  (+/-19.8) | 29.7  (+/-12.4) | 11.7  (+/-4.2) | 28.7  (+/-1.56) | 30.5  (+/-2.69) | 8.8  (+/-0.88) |
| SK-N-SH | 23.7  (+/-6.1) | 12.5  (+/-3.1) | 3.6  (+/-1.6) | 46.8  (+/-2.12) | 45.9  (+/-3.04) | 30.7  (+/-1.41) |
| K562 | 28.2  (+/-2.5) | 8.8  (+/-2.8) | No signal detected | 84.85  (+/-0.07) | 27.0  (+/-1.41) | Not done |

**Table S1**. Percent RFP-positive cells at different time points (post-transfection) as determined by flow cytometry. Cells were harvested by standard trypsinization (see Methods), resuspended in 1xPBS supplemented with 1% FBS, and analyzed on a BD Accuri C6 flow cytometer using CFlow Plus software. The filter used to measure RFP was 675 nm LP. Values represent averages of duplicate samples of PcTF-transfected cells. Standard deviations are shown in parenthesis.

| Gene Symbol | Transcript | Forward Primer (5’-..) | Reverse Primer (5’-..) | LNA probe |
| --- | --- | --- | --- | --- |
| ATF3 | NM_001030287 | aaggattttcagcaccttgc | gatggcagaagcactcacttc | 13 |
| CADM1 | NM_001098517 | gcgcatgtcattagcatctc | ctcggcagcactacactcg | 16 |
| CASZ1 | NM_001079843 | caacaacaacctggtgaacg | ttcggaactcgcagtgct | 54 |
| CDH1 | NM_004360 | ggtctgtcatggaaggtgct | gatggcggcattgtaggt | 5 |
| CDKN2A | NM_000077 | gtggacctggctgaggag | ctttcaatcggggatgtctg | 34 |
| CLU | NM_001831 | gggaccagacggtctcag | cgtacttacttccctgattggac | 1 |
| HK2 | NM_000189 | tccttccctgaaccttttcc | cagatttcaagagacatgacattagc | 22 |
| HOXA6 | NM_024014 | gcagcggatgaactcctg | ggttgaagtggaactccttctc | 47 |
| HOXB4 | NM_024015 | ctggatgcgcaaagttcac | agcggttgtagtgaaattcctt | 62 |
| IRF8 | NM_002163 | gaggtggtccaggtcttcg | cggccctggctgttatag | 20 |
| LAMB3 | NM_001017402 | cccagtttgctttgctgtg | gggcaaaacacaagaggaag | 41 |
| RUNX3 | NM_004350 | ggcctctccatgccttct | aggagggaagaaactacaaggac | 38 |
| TRIM22 | NM_006074 | tcacttctgagaatttaactttcgtt | ggctggtctccacaaatga | 17 |
| WT1 | NM_001198551 | agggctctgaggattgtgc | cgaaggtgaccgtgctgta | 37 |
| PcTF (mCherry) | n/a | cctgaagggcgagatcaag | ttgacctcagcgtcgtagtg | 41 |

**Table S2**. Primers and probes were designed to analyze, via real time quantitative PCR, the expression of genes that were predicted as targets of the synthetic chromatin protein. The HUGO Gene Symbol and RNA transcript ID is shown for each gene. The Roche Assay Design Center tool (<http://www.roche-applied-science.com/sis/rtpcr/upl/index.jsp?id=UP030000>) was used to choose each forward primer, reverse primer, and Locked nucleic acid (LNA) probe from the Roche Universal Probe Library (UPL).

| Target | Forward primer (5’..) | Reverse primer (5’..) | Amplicon size (bp) |
| --- | --- | --- | --- |
| CASZ1 P1 | ctggattcacacataatgactgtt | cacgtcgagcacagagaga | 101 |
| CASZ1 P2 | acaagtgtctctcttgggagt | gtcaagttcaccaacccgga | 102 |
| CASZ1 P3 | atcaggcctaccccacttct | ggctgttggagacgttgaga | 105 |
| CASZ1 P4 | ggagcctaatctgcaaggtgt | gcatcccggaagatgcttgt | 100 |
| CASZ1 P5 | ggtgactttccaagtccggg | ctgggagcgaaaaatccggg | 107 |
| GAPDH | tactagcggttttacgggcg | tcgaacaggaggagcagagagcga | 166 |

**Table S3**. Primers used for the ChIP-PCR assay (Figure 4).

SUPPLEMENTAL REFERENCES

1. Heinz S, Benner C, Spann N, Bertolino E, Lin YC, Laslo P, Cheng JX, Murre C, Singh H, Glass CK. (2010) Simple combinations of lineage-determining transcription factors prime cis-regulatory elements required for macrophage and B cell identities. Mol Cell. 38:576-89.
2. Liu T, Ortiz J, Taing L, Meyer C, Lee B, Zhang Y, Shin H, Wong S, Ma J, Lei Y, et al. (2011) Cistrome: an integrative platform for transcriptional regulation studies. Genome Biology. 12: R83.
